# Downregulated *ALDH2* Contributes to Tumor Progression and Targeted Therapy Resistance in Human Metastatic Melanoma Cells

**DOI:** 10.3390/cells14120913

**Published:** 2025-06-17

**Authors:** Zili Zhai, Takeshi Yamauchi, Karenna Sandoval, Kira Villarreal, Man Wai Charlotte Kwong, Emily J. Swanson, Aik Choon Tan, Mayumi Fujita

**Affiliations:** 1 Department of Dermatology, University of Colorado Anschutz Medical Campus, Aurora, CO 80045, USA; zili.zhai@cuanschutz.edu (Z.Z.); takeshi.yamauchi@cuanschutz.edu (T.Y.); kansandoval@salud.unm.edu (K.S.); kiravillarreal22@gmail.com (K.V.); manwaicha.kwong@cuanschutz.edu (M.W.C.K.); e.swanson2020@gmail.com (E.J.S.); 2 Department of Oncological Sciences, Huntsman Cancer Institute, University of Utah, Salt Lake City, UT 84112, USA; aikchoon.tan@hci.utah.edu; 3 Department of Biomedical Informatics, Huntsman Cancer Institute, University of Utah, Salt Lake City, UT 84112, USA; 4 Department of Veterans Affairs Medical Center, VA Eastern Colorado Health Care System, Aurora, CO 80045, USA; 5 Department of Immunology and Microbiology, University of Colorado Anschutz Medical Campus, Aurora, CO 80045, USA

**Keywords:** acetaldehyde, aldehyde dehydrogenase 2, drug resistance, MAPK/ERK, melanoma, targeted therapy

## Abstract

Aldehyde dehydrogenase 2 (ALDH2) is a crucial detoxifying enzyme that eliminates toxic aldehydes. ALDH2 deficiency has been linked to various human diseases, including certain cancers. We have previously reported *ALDH2* downregulation in human melanoma tissues. Here, we further investigated the biological significance of *ALDH2* downregulation in this malignancy. Analysis of TCGA dataset revealed that low *ALDH2* expression correlates with poorer survival in metastatic melanoma. Examination of human metastatic melanoma cell lines confirmed that most had *ALDH2* downregulation (*ALDH2*-low) compared to primary melanocytes. In contrast, a small subset of metastatic melanoma cell lines exhibited normal *ALDH2* levels (*ALDH2*-normal). CRISPR/Cas9-mediated *ALDH2* knockout in *ALDH2*-normal A375 cells promoted tumor growth and MAPK/ERK activation. Given the pivotal role of MAPK/ERK signaling in melanoma and cellular response to acetaldehyde, we compared A375 with *ALDH2*-low SK-MEL-28 and 1205Lu cells. *ALDH2*-low cells were intrinsically resistant to BRAF and MEK inhibitors, whereas A375 cells were not. However, A375 cells acquired resistance upon *ALDH2* knockout. Furthermore, melanoma cells with acquired resistance to these inhibitors displayed further ALDH2 downregulation. Our findings indicate that *ALDH2* downregulation contributes to melanoma progression and therapy resistance in *BRAF*-mutated human metastatic melanoma cells, highlighting ALDH2 as a potential prognostic marker and therapeutic target in metastatic melanoma.

## 1. Introduction

Aldehyde dehydrogenase (ALDH) 2 is one of 19 functional ALDH isoforms responsible for detoxifying endogenous and exogenous aldehydes [1]. It is best known for breaking down acetaldehyde (AcAH), an intermediate product of ethanol (EtOH) metabolism [2]. ALDH2 also oxidizes reactive lipid peroxidation byproducts, such as malondialdehyde and 4-hydroxy-2-nonenal, helping to mitigate redox imbalance during metabolism and aging [3]. In addition to its dehydrogenase activity, ALDH2 exhibits other enzymatic functions, including reductase activity that converts nitroglycerin to nitric oxide, an essential regulator of cardiac physiology [4]. Due to its diverse physiological and detoxifying activities, ALDH2 deficiency has been associated with a wide range of diseases, including cardiovascular and neurodegenerative disorders, alcohol-related liver disease, and cancer [5].

ALDH2 is widely expressed across human tissues [6], with the highest levels in the liver and significant abundance in the skin [7]. While its detoxification role in the liver is well established, its biological function in the skin remains unknown. As the body’s outermost barrier, the skin is exposed to reactive aldehydes from both endogenous metabolism and external sources [8]. Chronic EtOH administration has been reported to induce skin hyperpigmentation in *Aldh2*-deficient mice [9], suggesting a role for ALDH2 in skin biology and melanocyte physiology. In humans, alcohol consumption, particularly alcohol use disorders, has been linked to cutaneous changes and poses a risk of developing skin cancer [10]. Epidemiological evidence further supports a positive association between alcohol consumption and cutaneous melanoma [11,12]. Moreover, publicly available datasets reveal significantly downregulated *ALDH2* gene expression in human melanoma tissues compared to normal skin [8]. In vitro studies showed that *ALDH2* overexpression suppresses melanoma cell proliferation, migration, and invasion, suggesting its potential as a therapeutic target [13]. Based on these observations, we hypothesize that *ALDH2* downregulation in melanoma contributes to tumor progression.

The mitogen-activated protein kinase (MAPK)/extracellular signal-regulated kinase (ERK) signaling is a frequently mutated oncogenic pathway in melanoma, with approximately 50% of patients harboring *BRAF* mutations, most commonly V600E, which renders tumors sensitive to selective BRAF and MAPK kinase (MEK) inhibitors [14,15]. The introduction of targeted therapies, including BRAF inhibitors (e.g., vemurafenib (VEM) and dabrafenib) and MEK inhibitors (e.g., trametinib (TRA) and cobimetinib), has marked a significant therapeutic advancement for patients with *BRAF*-mutated melanoma over the past decade [15,16,17]. Although targeted therapy, together with immunotherapy, has significantly transformed the clinical management of melanoma, the development of acquired resistance remains a significant challenge. Under targeted therapy, most patients experience relapse within six months due to the emergence of acquired resistance [15,17,18]. As such, elucidating the mechanisms underlying MAPK/ERK pathway reactivation is critical for improving the efficacy of targeted treatments. Notably, EtOH, as well as AcAH and reactive oxygen species (ROS) generated during EtOH metabolism, can activate MAPK signaling, a central mediator of EtOH-related pathophysiological processes [19,20,21]. This observation prompts a strong interest in exploring the potential role of *ALDH2* downregulation in therapeutic resistance.

In this study, we demonstrate that decreased *ALDH2* gene expression in metastatic melanoma correlates with worse patient survival. Knocking out *ALDH2* in the A375 human metastatic melanoma cell line, which normally expresses ALDH2, enhances MAPK/ERK activation and promotes tumor growth. Additionally, *ALDH2* expression levels in *BRAF*-mutated metastatic melanoma cells influence cell sensitivity to BRAF and MEK inhibitors, with low *ALDH2* expression associated with both intrinsic and acquired resistance. Our findings highlight the critical role of ALDH2 as a detoxifying enzyme in melanoma, affecting both targeted therapy response and clinical outcomes.

## 2. Materials and Methods

### 2.1. Chemicals

BRAF inhibitor VEM, MEK inhibitor TRA, and ALDH2 inhibitor daidzin were from Selleck Chem (Houston, TX, USA). AcAH and ALDH2 activator Alda-1 were from Sigma (St. Louis, MO, USA). Other chemicals and reagents are indicated elsewhere.

### 2.2. Bioinformatics Analysis

Clinical data from 331 adult melanoma patients were obtained from the supplemental materials of The Cancer Genome Atlas (TCGA) dataset published in 2015 [22]. Of these, 286 patients, including 42 with primary melanoma and 244 with metastatic melanoma, had complete pathoclinical information. Normalized RNA-seqV2 level 3 gene expression data for *ALDH2* (RSEM values) of 244 metastatic melanoma patients were retrieved from the cBioPortal database [23,24]. The Swedish dataset GSE65904 [25], comprising 210 metastatic melanoma cases with complete clinical data, was also retrieved from the NCBI Gene Expression Omnibus (GEO) database for patient survival analysis. These two datasets were selected to assess the correlation between *ALDH2* expression and patient survival, given their large sample sizes and the availability of both genome-wide molecular data and clinical information in metastatic melanoma.

In addition, *ALDH2* gene expression data of Cancer Cell Line Encyclopedia (CCLE) human metastatic melanoma cell lines were downloaded from the Gene Expression and Mutations in Cancer Cell Lines (GEMiCCL) database [26].

### 2.3. Cell Culture and AcAH Treatment

Human metastatic melanoma and immortalized human melanocyte cell (PIG1) lines were originally from the American Type Culture Collection (Manassas, VA, USA). Melanoma cells were grown in RPMI 1640 supplemented with 10% fetal bovine serum. PIG1 cells were grown in Medium 254 (ThermoFisher Scientific, Grand Island, NY, USA) supplemented with 1% human melanocyte growth supplement (ThermoFisher Scientific) and 5% fetal bovine serum. Normal human dermal fibroblasts were from LifeLine Cell Technology (Frederick, MD, USA). Human neonatal epidermal keratinocytes, human neonatal epidermal melanocytes, darkly pigmented donor, and human neonatal epidermal melanocytes, lightly pigmented donor, were from ThermoFisher Scientific. These primary human skin cells were grown in a culture medium as recommended by the providers. Cells were monitored monthly for mycoplasma contamination.

Ice-cold AcAH was added into the culture medium, and culture vessels were immediately sealed tightly with a sterile film to minimize AcAH evaporation [20]. The tips used for pipetting AcAH were pre-chilled in the freezer to aid in drawing up. Cells were incubated at 37 °C in a 5% CO_2_ incubator.

### 2.4. Quantitative RT-PCR

Total RNA was isolated using the RNeasy Plus Mini kit (Qiagen, Hilden, Germany) and reverse transcribed using the iScript cDNA synthesis kit (Bio-Rad, Hercules, CA, USA). qPCR was performed with the PowerUp SYBR Green PCR Master Mix (Applied Biosystems, Foster City, CA, USA) on the AriaMx Real-time PCR System (Agilent, Santa Clara, CA, USA). The primers for *ALDH2*, *ATF4*, *IL1B*, *MITF*, *MITF-M*, *MYC*, *ALDH1A1*, *ALDH1A3*, *ALDH1B1*, and *GAPDH* are listed in Supplemental Table S1.

### 2.5. Western Blot

Cells were rinsed twice in ice-cold phosphate-buffered saline and lysed in RIPA buffer (Sigma) containing 1% (*v*/*v*) Halt protease and phosphatase inhibitor cocktail (Thermo Scientific, Rockford, IL, USA). Cell lysates were separated on 4–15% Mini-PROTEAN TGX precast gels (Bio-Rad), followed by electrotransfer onto PVDF membranes. After blocking with 5% nonfat milk, the immunoblots were incubated with primary antibodies and then a horseradish peroxidase-conjugated secondary antibody (Sigma). The primary antibodies included mouse anti-ALDH2 (Invitrogen, Rockland, IL, USA), p-p38α MAPK (Tyr182), p38α MAPK, p-ATF2 (Thr71) (Santa Cruz Biotechnology, Santa Cruz, CA, USA), and p-AKT (Ser473) (Cell Signaling Technology, Danvers, MA, USA), and rabbit antibodies against ATF2 (Selleck Chem), phospho-p44/42 MAPK (ERK1/2) (Thr202/Tyr204), p44/42 MAPK (ERK1/2), phospho-SAPK/JNK (Thr183/Tyr185), SAPK/JNK, AKT, cyclophilin A (CyPA), and GAPDH (Cell Signaling Technology). Signals were visualized by SuperSignal West Femto Maximum Sensitivity Substrate (Thermo Scientific) and analyzed using the Odyssey imaging system (LI-COR, Lincoln, NE, USA). The band densities were quantified using the ImageJ software (version 1.52a, NIH, Bethesda, MD, USA).

### 2.6. ALDH2 Knockout (KO)

*ALDH2*-KO A375 and HT-144 melanoma cells were generated using the CRISPR-Cas9 method. *ALDH2* CRISPR guide RNA (CCCGCTCGATCAGATCGGCC) and plasmid control pSpCas9 BB-2A-Puro (PX459) v2.0 were from GenScript (Piscataway, NJ, USA). The single gRNA was designed to target exon 3 in the *ALDH2* genome. Cells were transfected with 4 µg/well of plasmids in a 6-well plate overnight using Lipofectamine 2000 reagent (Invitrogen, Waltham, MA, USA) in OPTI-MEM1 reduced serum medium (Gibco, Grand Island, NY, USA). *ALDH2*-KO clones were selected by adding 1 μg/mL of puromycin (Sigma), and subsequently, single-cell clones were picked.

### 2.7. ALDH2 Overexpression (OE)

*ALDH2*-OE in 1205Lu and SK-MEL-28 melanoma cells was generated by transfecting cells with pCMV6-*ALDH2* (NM_000690) or pCMV6-Entry as a vector control (OriGene Technologies, Rockville, MD, USA). After overnight transfection in a 24-well plate with Lipofectamine 2000 in antibiotic-free complete medium, stable *ALDH2*-OE clones were selected with G418 sulfate (Gibco) up to 800 μg/mL.

### 2.8. ALDH2 Activity Assay

Mitochondrial ALDH2 activity was determined using a commercial assay kit from Abcam (Waltham, MA, USA) and expressed as mOD/min. The assay does not measure cytosolic ALDH1 activity.

### 2.9. AcAH Assay

Cells were seeded in 6-well plates in Gibco phenol red-free RPMI 1640 (Thermo Scientific) containing 10% fetal bovine serum and allowed to attach for 2 h. The plate wells were then sealed with sterile film to confine AcAH in the culture medium and headspace, and the cells were incubated at 37 °C for an additional 20 h. AcAH in culture medium was quantified using the EnzyChrom AcAH assay kit (BioAssay Systems, Hayward, CA, USA). A culture medium without cells was used as background control.

### 2.10. Cellular ROS Assay

Cells were incubated overnight in the dark, clear-bottom 96-well plates. Baseline cellular ROS production was quantified using DCFDA Cellular ROS Assay kit or Cellular ROS Assay kit (Red) (Abcam) according to the manufacturer’s microplate assay instructions.

### 2.11. Cellular Energy Metabolism Assay

Oxygen consumption rate (OCR) and extracellular acidification rate (ECAR) of A375 and 1205Lu cells were measured with the Seahorse XF Cell Mito Stress Test kit (Agilent Technologies, Cedar Creek, TX, USA). Briefly, 1 × 10^4^ cells were seeded into a Seahorse XF96 cell culture microplate and incubated overnight. Freshly prepared modulators of mitochondrial respiration, oligomycin (final 2.5 μM), carbonyl cyanide-4-(trifluoromethoxy)phenylhydrazone (FCCP; 2 μM), a mix of rotenone/antimycin A (0.5 μM each) (Agilent Technologies), and 2-deoxyglucose (50 mM) (Sigma) were sequentially injected into each well and mitochondrial respiration was detected on the Agilent Seahorse XF96 analyzer. Seahorse XF96 Wave software (version 2.6.3.5) was used to analyze the results.

### 2.12. Tumor Formation In Vivo

One million A375 cells were suspended in high-concentration Matrigel Matrix (Corning, Bedford, MA, USA) diluted 1:3 with ice-cold RPMI-1640 and injected subcutaneously into both flanks of 7-week-old athymic nu/nu mice from Jackson Laboratories (Bar Harbor, ME, USA). Tumor growth was monitored every 2–3 days with an electronic digital caliper, and tumor volume was calculated according to the formula: tumor volume (mm^3^) = longest diameter × shortest diameter^2^/2. The experimental manipulations were approved by the Institutional Animal Care and Use Committee of the University of Colorado Anschutz Medical Campus under protocol number 00282, with an initial approval date of 10 September 2017 and subsequent renewals in 2020 and 2023.

### 2.13. Interleukin (IL)-1β Secretion

Around 80% confluent 1205Lu cells in 12-well transparent plates were added with fresh medium and continued to culture overnight. Culture supernatants were harvested for analysis of secreted IL-1β using a human IL-1β ELISA kit (R&D Systems, Minneapolis, MN, USA).

### 2.14. Cell Viability Assay

Melanoma cells at 1.5 × 10^3^ cells/well in 96-well transparent plates were treated with one dose of VEM ranging from 0.01 to 10 μM or TRA from 0.1 nM to 10 μM for 72 h. In the second set of experiments, 1.5 × 10^3^ melanoma cells were treated with one dose of 1 µM VEM and/or 0.1 µM TRA for 48 h. Cell growth inhibition was analyzed using the CellTiter 96 Aqueous One Solution Cell Proliferation assay kit (Promega, Madison, WI, USA).

### 2.15. Generation of Acquired Resistance

The development of VEM- and TRA-resistant melanoma cells with a stepwise dose-escalation strategy has been previously described [27].

### 2.16. Statistical Analysis

GraphPad Prism 9 (GraphPad Software, La Jolla, CA, USA) was used for statistical analysis. Kaplan-Meier method was used to create survival curves, and the log-rank test was used for statistical differences between two patient groups. Experimental numerical data are expressed as mean ± standard deviation (SD). The two-tailed Student’s *t*-test was used to compare two groups, while a one-way ANOVA with Sidak’s multiple comparisons test was used for comparison among more groups. A *p*-value < 0.05 was considered statistically significant.

## 3. Results

### 3.1. Low ALDH2 Expression Correlates with Worse Overall Survival in Metastatic Melanoma

**Figure 1:**
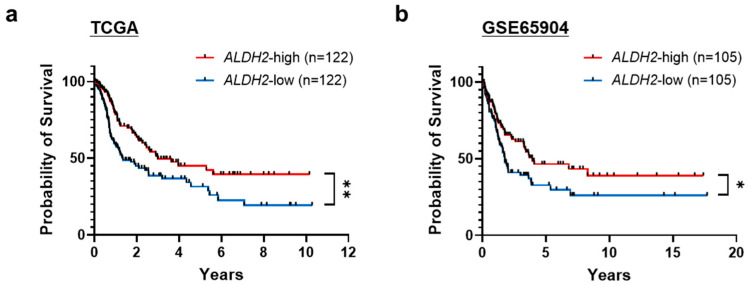
Relationship between aldehyde dehydrogenase 2 (*ALDH2*) gene expression and metastatic melanoma patient survival. Kaplan-Meier survival curves of TCGA (**a**) and Swedish dataset GSE65904 (**b**) metastatic melanoma patients. Patients with complete clinicopathological information were dichotomized into *ALDH2*-high and *ALDH2*-low expression groups based on their normalized RNA-seq expression levels of the *ALDH2* gene. * *p* < 0.05 and ** *p* < 0.01, log-rank test.

Analysis of multiple independent GEO datasets confirmed significantly downregulated *ALDH2* mRNA expression in melanoma tissues [8]. To understand its clinical relevance, we examined the correlation between *ALDH2* gene expression levels and overall survival in metastatic melanoma patients from TCGA with complete pathoclinical data. Our analysis revealed a significant correlation between low *ALDH2* expression and worse overall survival in these patients (Figure 1a). Another publicly available dataset of metastatic melanoma tumors from the Swedish cohort further validated this association with melanoma patient survival (Figure 1b). These results demonstrate that downregulated *ALDH2* in melanoma is correlated with poorer patient survival.

### 3.2. Melanoma Cells Are Classified as ALDH2-Normal or ALDH2-Low, Compared to Normal Skin Cells

**Figure 2:**
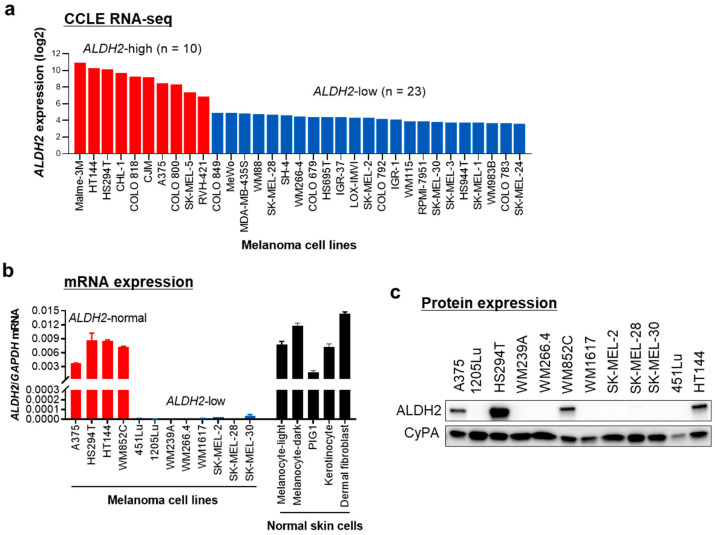
*ALDH2* is expressed at low levels in most human metastatic melanoma cell lines. (**a**) *ALDH2* gene expression data of Cancer Cell Line Encyclopedia (CCLE) human melanoma cell lines were obtained from the Gene Expression and Mutations in Cancer Cell Lines (GEMiCCL) database. (**b**) qRT-PCR analysis of *ALDH2* mRNA levels in 12 human metastatic melanoma cell lines and 5 normal skin cell types. *GAPDH* served as an internal control. Data are presented as mean ± SD (n = 3). (**c**) Western blot analysis of ALDH2 protein levels in the 12 human metastatic melanoma cell lines shown in (**b**). Cyclophilin A (CyPA) was used as a loading control.

Next, we examined *ALDH2* expression in human metastatic melanoma cell lines. Analysis of the CCLE dataset revealed that among 33 metastatic melanoma cell lines, 70% exhibited low *ALDH2* expression, whereas the remaining 30% showed relatively high expression levels (Figure 2a). We then investigated 12 human metastatic melanoma cell lines and 5 normal human skin cell types, including primary melanocytes (light and dark pigmented), keratinocytes, fibroblasts, and the immortalized melanocyte PIG1. Similar to the CCLE dataset, two-thirds of human metastatic melanoma cell lines showed reduced *ALDH2* expression levels compared to normal skin cells, while one-third displayed normal expression. Based on these findings, we categorized melanoma cell lines into *ALDH2*-normal and *ALDH2*-low groups (Figure 2b). The differences in *ALDH2* gene expression were consistently reflected at the protein level (Figure 2c and Figure S1), confirming the distinction between *ALDH2*-normal and *ALDH2*-low melanoma cells.

### 3.3. ALDH2-KO Impairs AcAH Detoxification and Alters Redox Balance and Cellular Energy Metabolism in ALDH2-Normal A375 Cells

**Figure 3:**
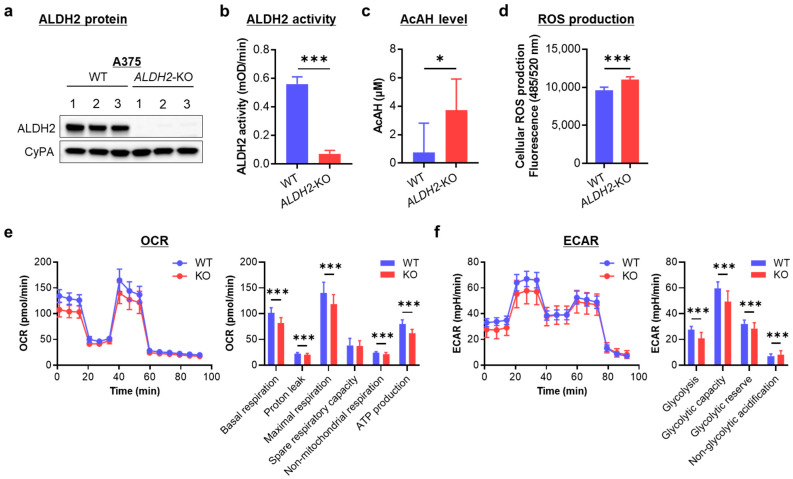
Effects of knocking out *ALDH2* on acetaldehyde (AcAH) and reactive oxygen species (ROS) production and cellular energy metabolism in A375 cells. (**a**) *ALDH2* knockout (KO) was confirmed by Western blot in three single-cell clones. WT, wild-type. (**b**) Mitochondrial ALDH2 activity. (**c**) AcAH levels in the culture medium of cells. (**d**) Intracellular ROS levels were detected using a DCFDA cellular ROS assay kit. (**e**) Cellular oxygen consumption rate (OCR) was measured with the Seahorse XF Cell Mito Stress Test kit. Left: respiratory flux profile; right: quantification of respiratory parameters. (**f**) The extracellular acidification rate (ECAR) was measured with the Seahorse assay. The data are representative of 2–4 experiments and expressed as the mean ± SD (n = 3 (**b**), 7 (**c**), 12 (**d**) or 19 or 22 (**e**,**f**)). * *p* < 0.05 and *** *p* < 0.001.

Because *ALDH2* is downregulated in two-thirds of melanoma cells, we knocked out this gene in *ALDH2*-normal A375 cells to investigate the effects of *ALDH2* loss. Western blot analysis confirmed successful CRISPR/Cas9-mediated loss of ALDH2 expression in three single-cell clones (Figure 3a and Figure S2). With *ALDH2* gene KO, baseline ALDH2 enzymatic activity was significantly decreased (Figure 3b).

To assess the impact of low ALDH2 on AcAH metabolism, we quantified AcAH in the culture medium of A375 cells. Wild-type (WT) A375 cells released only trace amounts of AcAH. In contrast, AcAH levels were significantly elevated in the culture medium of *ALDH2*-KO A375 cells (Figure 3c), indicating impaired AcAH detoxification after *ALDH2* loss.

Given that impaired ALDH2 function and elevated AcAH levels lead to ROS generation and oxidative stress [21,28], we next measured baseline ROS levels after *ALDH2* loss. *ALDH2*-KO A375 cells exhibited a slight but significant increase in intracellular ROS compared to WT cells (Figure 3d), suggesting a role for ALDH2 in regulating AcAH metabolism and ROS production.

As a mitochondrial enzyme, ALDH2 participates in electron transfer activities and regulates cellular bioenergetics [29]. Reduced ALDH2 activity and elevated AcAH levels can disrupt metabolic homeostasis and contribute to mitochondrial dysfunction [30]. To determine whether ALDH2 reduction affects energy metabolism, we measured OCR and ECAR. OCR reflects mitochondrial respiration, including basal and maximal respiration (Figure 3e). Basal respiration comprises ATP-linked oxygen consumption and proton leak uncoupled from ATP production. Knocking out *ALDH2* significantly reduced both components of basal respiration. Maximal respiration comprises basal respiration and spare respiratory capacity. Spare respiratory capacity reflects the cell’s ability to meet increased energy demands beyond basal respiration. When *ALDH2* was knocked out, A375 cells reduced maximal respiration. However, this reduction primarily resulted from lower basal respiration rather than a decrease in spare respiratory capacity, indicating that without a metabolic challenge or a stressor, knocking out *ALDH2* reduces basal respiration but does not affect spare respiratory capacity. Additionally, *ALDH2* loss led to decreased non-mitochondrial respiration. These findings suggest suppressed oxygen consumption both within and outside the mitochondria following *ALDH2* loss.

On the other hand, ECAR is associated with lactate efflux and indicates glycolytic activity, cellular activation, and proliferation status. ECAR comprises glycolytic acidification linked to lactate production and non-glycolytic acidification arising from other cellular processes such as CO_2_ hydration. *ALDH2* deficiency reduced glycolytic parameters, as shown by decreased lactate-associated acidification, while slightly increasing non-glycolytic acidification (Figure 3f). These findings suggest that *ALDH2* loss alters cellular metabolic dynamics, potentially impacting cellular activation and proliferation.

Collectively, the findings highlight a crucial role for *ALDH2* in AcAH detoxification, redox homeostasis, and cellular energy metabolism in A375 cells.

### 3.4. ALDH2 Downregulation Promotes Melanoma Tumor Growth by Enhancing AcAH-Mediated MAPK/ERK Activation, Inflammation, and Glycolytic Reprogramming

**Figure 4:**
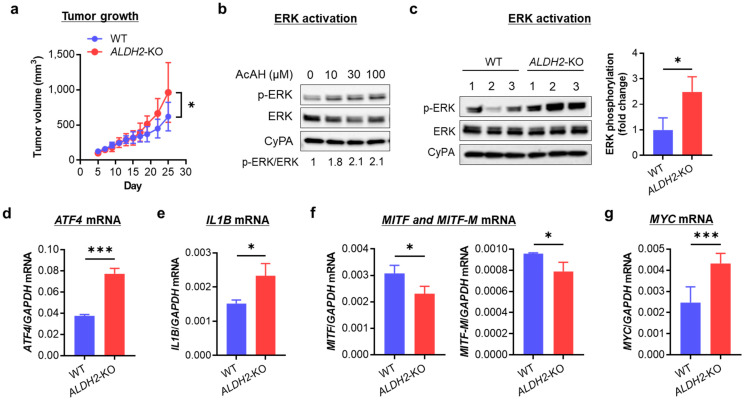
Effects of knocking out *ALDH2* on A375 tumor growth, ERK activation, and mRNA expression of *ATF4, IL1B, MITF,* and *MYC*. (**a**) Tumor growth curves of A375 cells injected subcutaneously in 8 female nude mice (4 for WT and 4 for *ALDH2*-KO). (**b**) Western blot of p-ERK in A375 cells treated with 0–100 μM AcAH for 1 h. Band densities of phosphorylated ERK were quantified and adjusted by those of total ERK at the same dose of AcAH. (**c**) Western blot of ERK phosphorylation in three single clones of WT and *ALDH2*-KO A375 cells (**left** panel). Band densities of phosphorylated ERK were quantified and adjusted by those of total ERK (**right** panel). (**d**) qRT-PCR of *ATF4* mRNA. (**e**) qRT-PCR of *IL1B* mRNA. (**f**) qRT-PCR of *MITF* and *MITF-M* mRNA. (**g**) qRT-PCR of *MYC* mRNA. The data are expressed as the mean ± SD (n = 8 (tumors, (**a**)) or 3 (**c**–**g**)). * *p* < 0.05 and *** *p* < 0.001.

To investigate the biological effects of *ALDH2*-KO, we evaluated A375 tumor growth by injecting nude mice with WT and *ALDH2*-KO A375 cells. Tumors derived from *ALDH2*-KO cells grew significantly faster than those from WT A375 cells (Figure 4a), indicating that *ALDH2* loss promotes tumor growth. To explore the underlying mechanism, we evaluated MAPK signaling activation, a key regulator of melanoma biology.

We first assessed the phosphorylation of ERK, p38, and JNK in WT A375 cells following exposure to AcAH. Around 1 h after 5 mM AcAH exposure, the phosphorylation levels of ERK and JNK peaked, while p38 remained highly phosphorylated between 15 and 60 min (Figure S3). We then examined whether pharmacological manipulation of ALDH2 activity could alter AcAH-mediated MAPK activation in A375 cells. Cells were pretreated with the ALDH2 inhibitor daidzin or the activator Alda-1 for 2 h, followed by exposure to 5 mM AcAH for 1 h. While daidzin enhanced AcAH-mediated MAPK activation, Alda-1 pretreatment inhibited activation of all three MAPKs (Figure S4), suggesting that ALDH2 negatively regulates MAPK activation in human melanoma cells. Next, we selected a 1-h incubation time and treated A375 cells with physiological doses of AcAH (≤100 µM) [31,32], observing notable activation of MAPK/ERK (Figure 4b and Figure S5). Based on this, we focused on MAPK/ERK signaling and found that *ALDH2*-KO A375 cells exhibited increased baseline MAPK/ERK phosphorylation compared to WT cells (Figure 4c and Figure S6). These findings suggest that *ALDH2* deficiency promotes tumor growth, possibly through AcAH-mediated MAPK/ERK activation. This increase in MAPK/ERK phosphorylation was further validated in another *ALDH2*-KO cell line, HT-144 (Figure S7).

Melanoma cells exhibit IL-1β-driven autoinflammatory properties, partially regulated by the MAPK/ERK pathway and transcription factor ATF4 [27]. Based on these associations, we examined whether increased MAPK/ERK phosphorylation influences inflammatory signaling in *ALDH2*-KO A375 cells. Indeed, *ALDH2*-KO A375 cells showed significantly increased expression of *ATF4* (Figure 4d) and *IL1B* (Figure 4e) compared to WT cells. This upregulated *IL1B* expression following *ALDH2*-KO was confirmed in HT-144 cells (Figure S8a).

MAPK/ERK activation also affects the metabolic plasticity of tumor cells by suppressing oxidative phosphorylation and promoting glycolysis, thereby enhancing tumor cell survival [33]. To examine this metabolic shift, we analyzed the expression of two key transcription factors: MITF, which promotes oxidative phosphorylation, and MYC, which drives glycolysis [33]. *ALDH2*-KO A375 cells exhibited decreased *MITF* and *MITF-M* expression (Figure 4f) and increased *MYC* expression (Figure 4g), indicating a metabolic shift towards glycolysis after *ALDH2* downregulation. This pattern was similarly observed in *ALDH2*-KO HT-144 cells (Figure S8b).

Collectively, these findings suggest that *ALDH2* downregulation enhances the inflammatory phenotype and drives metabolic reprogramming through the MAPK/ERK signaling pathway, ultimately promoting tumor growth.

### 3.5. ALDH2-OE Alleviates AcAH, ROS, and IL-1β Production and Promotes Mitochondrial Respiration and a Metabolic Shift Toward Oxidative Phosphorylation in ALDH2-Low 1205Lu Cells

After establishing that *ALDH2* loss disrupts AcAH detoxification, redox homeostasis, and energy metabolism while promoting inflammation and glycolytic reprogramming in melanoma cells, we next overexpressed this gene in *ALDH2*-low 1205Lu cells to investigate the effects of its restoration.

**Figure 5:**
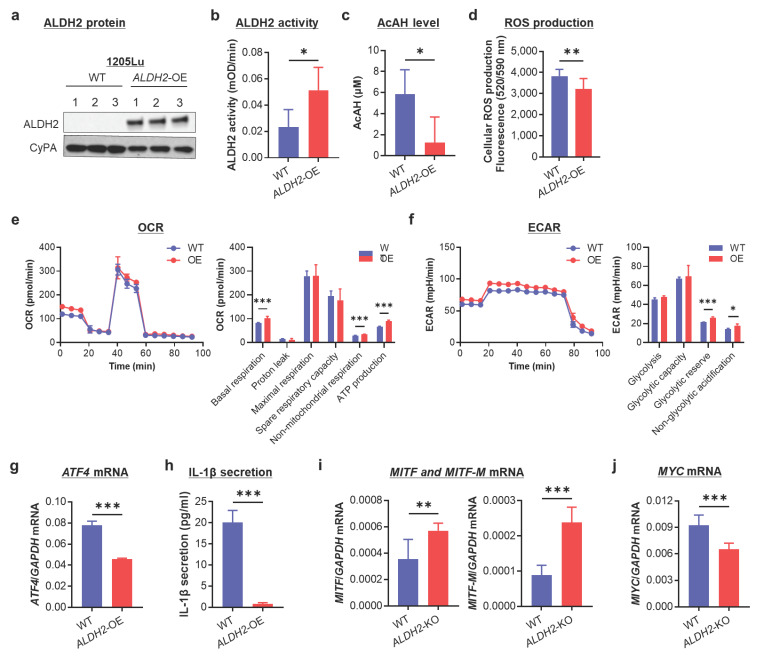
Effects of overexpressing *ALDH2* on AcAH and ROS production, mitochondrial respiration, IL-1β secretion, and *MITF*/*MYC* expression in 1205Lu cells. (**a**) Confirmation of *ALDH2* overexpression (OE) by Western blot. (**b**) Mitochondrial ALDH2 activity. (**c**) AcAH levels in culture medium. (**d**) Intracellular ROS levels were detected using a cellular ROS assay kit (Red). (**e**) Measurement of OCR. (**f**) Measurement of ECAR. (**g**) *ATF4* mRNA. (**h**) IL-1β secretion by ELISA. (**i**) *MITF* and *MITF-M* mRNA. (**j**) *MYC* mRNA. The data are representative or expressed as the mean ± SD (n = 5 (**b**), 4 (**c**), 10 (**d**), 4–6 (**e**,**f**), 3 (**g**), 9 (**h**) or 6 (**i**,**j**). * *p* < 0.05, ** *p* < 0. 01, and *** *p* < 0.001.

OE of *ALDH2* in 1205Lu cells was confirmed by increased ALDH2 protein expression levels (Figure 5a and Figure S9) and enzymatic activity (Figure 5b). Compared to WT 1205Lu, *ALDH2*-OE cells exhibited reduced extracellular AcAH release (Figure 5c) and intracellular ROS generation (Figure 5d). Measurement of the OCR as a readout of mitochondrial respiration showed that overexpressing *ALDH2* increased basal respiration (ATP production) and non-mitochondrial respiration (Figure 5e). Further ECR analysis revealed enhanced glycolytic reserve and non-glycolytic acidification (Figure 5f). These findings suggest that *ALDH2*-OE enhances mitochondrial function and promotes overall cellular energy metabolism.

1205Lu is a typical melanoma cell line with constitutive secretion of IL-1β [34]. We demonstrated that *ATF4* expression (Figure 5g) and IL-1β secretion (Figure 5h) were significantly reduced upon *ALDH2*-OE in 1205Lu cells. Furthermore, *ALDH2*-OE resulted in increased *MITF* and *MITF-M* expression (Figure 5i), accompanied by decreased *MYC* expression (Figure 5j). These findings suggest that *ALDH2*-OE attenuates the inflammatory phenotype and promotes a metabolic shift toward oxidative phosphorylation.

Collectively, these findings further indicate that ALDH2 plays a role not only in AcAH detoxification but also in regulating cellular energy metabolism, auto-inflammation, and metabolic reprograming in melanoma cells.

### 3.6. Melanoma Cells with Low ALDH2 Expression Harbor Intrinsic Resistance to MAPK/ERK Signaling Inhibition

Given that *ALDH2* downregulation leads to increased MAPK/ERK activation and a metabolic shift toward glycolysis, both of which confer resistance to targeted therapy [33], we investigated the relationship between *ALDH2* expression levels and drug sensitivity to targeted therapy.

**Figure 6:**
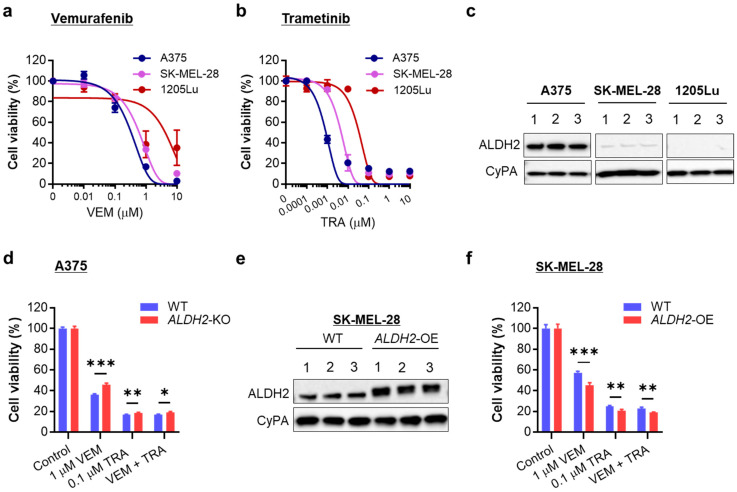
Relation of *ALDH2* expression levels to sensitivity to BRAF and MEK inhibitors. (**a**) MTS proliferation assay of three *BRAF*-mutated melanoma cell lines treated with a single dose of vemurafenib (VEM) at the indicated doses for 72 h. (**b**) MTS proliferation assay of three melanoma cell lines treated with a single trametinib (TRA) dose at the indicated doses for 72 h. (**c**) Western blot analysis of ALDH2 levels in A375, SK-MEL-28, and 1205Lu cells (3 cell lysate samples each). (**d**) MTS proliferation assay of WT and *ALDH2*-KO A375 cells treated with one dose of 1 µM VEM and/or 0.1 µM TRA for 48 h. (**e**) *ALDH2*-OE in SK-MEL-28 cells was confirmed by Western blot. (**f**) MTS proliferation assay of WT and *ALDH2*-OE SK-MEL-28 cells treated with one dose of VEM and/or TRA for 48 h. The data are expressed as the mean ± SD (n = 3 (**a**,**b**) or 4 (**d**,**f**). * *p* < 0.05, ** *p* < 0.01, and *** *p* < 0.001.

We compared drug sensitivity among *ALDH2*-normal A375 and *ALDH2*-low SK-MEL-28 and 1205Lu, all of which carry *BRAF^V600E^* mutations. Sensitivity to VEM followed the trend A375 > SK-MEL-28 > 1205Lu (Figure 6a). A similar pattern was observed with the MEK inhibitor TRA (Figure 6b), aligning with their ALDH2 expression levels (Figure 6c and Figure S10). These findings suggest that *ALDH2*-low melanoma cells display intrinsic resistance to MAPK/ERK pathway inhibition.

To further validate this, we evaluated changes in VEM and TRA sensitivity in A375 upon *ALDH2*-KO. As expected, *ALDH2*-KO A375 cells showed greater resistance to proliferation inhibition by physiological doses of VEM, TRA, and combination than *ALDH2*-WT cells (Figure 6d). Conversely, *ALDH2*-OE SK-MEL-28 cells (Figure 6e and Figure S11) became more susceptible to VEM, TRA, and combination than WT cells (Figure 6f). Increased drug sensitivity resulting from *ALDH2* upregulation was also demonstrated in 1205Lu cells (Figure S12).

These findings demonstrate that ALDH2 expression levels influence drug sensitivity to targeted therapy in *BRAF*-mutated metastatic melanoma cells, with low ALDH2 conferring resistance to MAPK/ERK-targeted therapy.

### 3.7. Acquired Resistance in Melanoma Cells Is Associated with Reduced ALDH2 Expression

**Figure 7:**
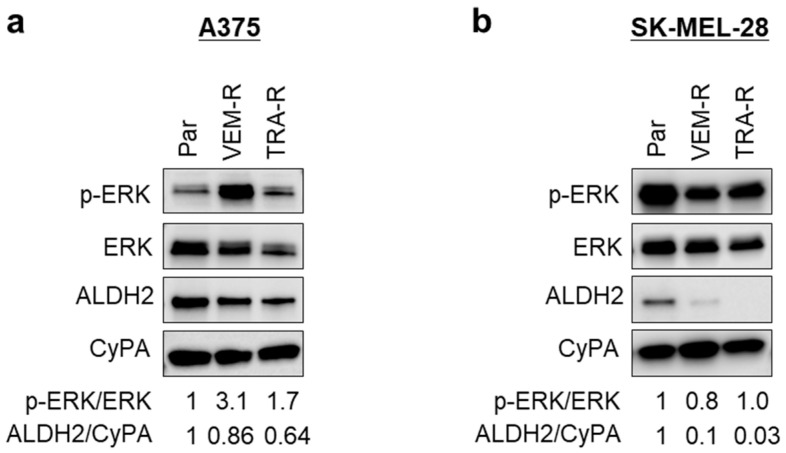
ALDH2 expression in VEM- and TRA-resistant melanoma cells. (**a**) Western blot analysis of ERK phosphorylation and ALDH2 expression in parental (Par), VEM-resistant (VEM-R), and TRA-resistant (TRA-R) A375 cells. The band densities of p-ERK and ALDH2 were quantitated and normalized. (**b**) Western blot analysis of ERK phosphorylation and ALDH2 expression in Par, VEM-R, and TRA-R SK-MEL-28 cells. The data are representative of 2–3 experiments.

Having established a correlation between *ALDH2* expression levels and drug sensitivity, we next examined ALDH2 expression in melanoma cells with acquired resistance to MAPK/ERK pathway inhibition. We utilized VEM- and TRA-resistant A375 and SK-MEL-28 cells, generated using previously published selection strategies [27]. Both resistant A375 (Figure 7a and Figure S13) and resistant SK-MEL-28 (Figure 7b and Figure S13) cells, which exhibited sustained MAPK/ERK activation under drug pressure, showed reduced ALDH2 expression compared to their corresponding parental counterparts. These findings suggest that ALDH2 downregulation contributes to the development of acquired resistance to MAPK/ERK pathway inhibitors.

Together, our findings highlight the importance of ALDH2 in melanoma biology, linking its downregulation to tumor progression, MAPK/ERK activation, metabolic reprogramming, resistance to targeted therapy, and worse patient outcomes.

## 4. Discussion

ALDH2 plays a crucial role in oxidizing toxic aldehydes from both exogenous and endogenous precursors, including those generated from endoplasmic reticulum stress, oxidative stress, hypoxia, and other cellular stressors, thereby mitigating their damaging effects [5,35,36]. Consequently, genetic, pharmacological, or biological downregulation of ALDH2 is expected to yield substantial effects, particularly disrupting alcohol metabolism and increasing the toxicity of xenobiotic and metabolic aldehydes. Our present study provides compelling evidence for the functional significance of *ALDH2* downregulation in melanoma biology and its impact on tumor progression and therapy resistance. By knocking out *ALDH2* in *ALDH2*-normal A375 cells, we demonstrated that *ALDH2* downregulation enhanced AcAH and ROS production, MAPK/ERK activation, tumor growth, and resistance to targeted therapy, underscoring its clinical relevance and association with poorer patient survival.

AcAH is a ubiquitous environmental toxin, and humans are exposed to AcAH through various routes, including alcohol consumption, food ingestion, inhalation, skin contact, and microbial metabolism [8]. EtOH-metabolizing enzymes are expressed in various tissues, enabling local AcAH production and contributing to both localized and systemic effects of alcohol consumption [37]. In individuals with alcohol use disorders, AcAH degradation is impaired, likely due to a primary enzymatic abnormality or secondary to liver damage, leading to elevated blood AcAH levels [38], with blood concentrations reaching 10 to 100 μM after consumption of 1.2 g/kg body weight of EtOH [31]. Even in nonalcoholic individuals, genetic variation can significantly influence AcAH accumulation. Following moderate alcohol intake (0.5 g/kg), ALDH2*1/*2 heterozygotes reached peak blood AcAH levels of approximately 86 μM, compared to around 2.5 μM in ALDH2*1/*1 WT individuals, underscoring the profound impact of *ALDH2* genotype on AcAH metabolism [32]. Once in the bloodstream, AcAH can readily diffuse into the skin [39]. Although skin AcAH exposure is generally low due to the presence of ALDH2 and other metabolizing enzymes [7,8,40], ALDH2 dysfunction has important pathological implications. *ALDH2* expression is significantly downregulated in most cancer types [41,42], including cutaneous melanoma [8]. Our analysis of multiple melanoma datasets in the Gene Expression Omnibus (GEO) revealed a progressive decrease in *ALDH2* expression from normal human skin to primary melanoma and further to metastatic melanoma [8]. Consistently, the majority of CCLE melanoma cell lines exhibited low *ALDH2* expression, with 2/3 of metastatic melanoma cell lines showing extremely low or undetectable levels compared to primary melanocytes. These findings suggest that low ALDH2 expression and activity may render melanoma cells more vulnerable to cytotoxic and genotoxic aldehydes, contributing to melanoma progression.

ALDH2 downregulation and dysfunction likely result from a complex interplay of genetic predisposition (e.g., polymorphisms and epigenetic regulation) and environmental or lifestyle factors such as pollution, alcohol intake, tobacco use, and high-fat diets [43]. The well-characterized ALDH2*2 allele (rs671) markedly influences enzyme activity: heterozygous individuals retain <20% activity, while homozygous carriers exhibit near-complete enzymatic inactivity [44]. When individuals with reduced ALDH2 activity consume EtOH, they accumulate significantly higher levels of AcAH in the blood compared to WT individuals [32], thereby facing an increased risk of developing alcohol-related cancers [45]. However, the role of *ALDH2* polymorphisms in melanoma remains inconclusive. The ALDH2*2 allele is prevalent in East Asians, who have a relatively low incidence of melanoma, and rare in Caucasians, who are at higher risk [46]. In our global analysis of melanoma datasets, we observed a strong positive correlation between WT *ALDH2* and melanoma incidence, while variant alleles showed an inverse relationship [47]. A similar trend was observed in alcohol consumption patterns: individuals with the WT allele tended to consume more alcohol, whereas carriers of the ALDH2*2 variant consumed less [47]. These findings suggest weak or little evidence for a direct causal link between *ALDH2* mutation and melanoma development. Notably, both TCGA and Swedish melanoma cohorts analyzed for survival outcomes in this study consisted primarily of individuals of European descent, in whom the rs671 variant is rare. Therefore, the observed differences in ALDH2 function are more likely attributable to altered gene expression rather than inherited genetic variants. The mechanisms governing *ALDH2* downregulation and inactivation in melanoma may involve multiple layers of regulation, including transcriptional control, mRNA stability, and epigenetic modulation, as previously reviewed [8,45].

To investigate the significance of *ALDH2* downregulation in melanoma biology, we knocked out *ALDH2* in *ALDH2*-normal A375 cells, resulting in a more aggressive tumor phenotype, supporting ALDH2 as a melanoma tumor suppressor. This finding aligns with reports of lung adenocarcinoma and hepatocellular carcinoma [48,49]. The accelerated tumor growth in *ALDH2*-KO A375 cells was, at least in part, attributed to elevated AcAH and ROS production. Notably, previous studies have shown that malignant human cells, such as CALU-1 (non-small-cell lung cancer cell line) [50], VA13 (hepatocellular carcinoma-derived cell line) [51], and HL60 (leukemia cell line) [52], can spontaneously release AcAH in vitro. AcAH is known to cause oxidative stress and inflammation by damaging DNA and proteins and forming macromolecule-centered radicals [28,39,53]. Additionally, ROS-mediated mitochondrial dysfunction is a well-established mechanism underlying EtOH-related pathological changes [54]. ROS production occurs during EtOH oxidation and is further amplified by ALDH2 inhibition and AcAH exposure [21,28,39,55]. Consistently, we observed that, even without any inducers or stressors, *ALDH2*-KO A375 cells produced higher basal levels of AcAH and ROS than WT cells. Both AcAH and ROS are recognized activators of MAPK signaling and contributors to tumorigenesis [19,28,56]. Indeed, we observed that a physiological concentration of AcAH at 10 μM was sufficient to activate MAPK/ERK phosphorylation in A375 cells in vitro. Therefore, it is reasonable to propose that the combinatorial effect of elevated basal levels of AcAH and ROS in *ALDH2*-KO cells contributes to MAPK/ERK activation. In this regard, ALDH2 may suppress tumor growth by curbing AcAH-mediated oxidative stress and MAPK/ERK activation.

Tumor-promoting inflammation is a hallmark of cancer. Certain metastatic melanoma cells exhibit features of an autoinflammatory state by constitutively releasing IL-1β through sustained activation of the inflammasome complex [34]. This tumor-driven IL-1β contributes to an inflammatory tumor microenvironment that fosters immunosuppression and supports tumor progression [34,57]. Additionally, tumor-intrinsic inflammasome activation and IL-1β secretion have been implicated in shaping treatment responses, particularly by promoting resistance to targeted therapy and other anticancer interventions [27,34,58]. In melanoma, IL-1β secretion is partially regulated by the MAPK/ERK pathway via the transcription factor ATF4 [27]. Notably, transgenic mice carrying the Aldh2*2 variant, which impairs ALDH2 enzymatic activity, show elevated Atf4 protein in the heart compared to WT littermates [59], suggesting a functional link between ALDH2 activity and the MAPK/ERK/ATF4/IL-1β signaling axis. In our study, *ALDH2*-KO in A375 cells enhanced MAPK/ERK pathway activation, accompanied by increased expression of *ATF4* and *IL1B.* In contrast, *ALDH2*-OE in 1205Lu cells suppressed *ATF4* expression and IL-1β secretion. These findings indicate that ALDH2 negatively regulates the inflammatory phenotype of melanoma cells, thereby influencing tumor growth and potentially modulating therapeutic response.

*ALDH2*-KO in A375 melanoma cells resulted in a reduction of both mitochondrial respiration, as measured by OCR, and glycolytic activity, as measured by ECAR. While the precise contribution of these metabolic changes to accelerated tumor growth remains to be fully elucidated, a shift in the MITF/MYC balance provides additional insight. This balance is a critical regulator of tumor heterogeneity, phenotypic plasticity, and drug resistance, particularly in melanoma with neural crest lineage characteristics [33,60]. *ALDH2*-KO resulted in downregulation of *MITF* and upregulation of *MYC*. This MITF-to-MYC transition may represent a key oncogenic program that supports tumor growth, promotes adaptive survival, and contributes to resistance to targeted therapy.

Given the alterations observed following *ALDH2*-KO in melanoma cells, such as activation of the MAPK/ERK pathway, enhanced inflammatory phenotypes, and a metabolic shift towards glycolysis, we examined how ALDH2 status influences the sensitivity of melanoma cells to MAPK/ERK inhibitors. *ALDH2*-low metastatic melanoma cells exhibited intrinsic resistance to BRAF and MEK inhibitors, a phenomenon confirmed in *ALDH2*-KO A375 cells. Conversely, *ALDH2*-OE in SK-MEL-28 and 1205Lu cells increased their sensitivity to these inhibitors. The relationship between *ALDH2* expression and resistance to targeted therapy was also evidenced in A375 and SK-MEL-28 cells with acquired resistance to BRAF/MEK inhibitors, both of which showed decreased ALDH2 expression. Notably, sustained MAPK/ERK activation in resistant cells appeared to suppress *ALDH2* expression, suggesting a potential feedback inhibitory loop between ALDH2 and MAPK/ERK signaling. This speculation is further supported by our observation that treatment of parental A375 and SK-MEL-28 cells with a single dose of VEM or TRA led to MAPK/ERK inactivation and ALDH2 upregulation (Figure S14). Although the mechanism by which MAPK/ERK signaling regulates ALDH2 expression remains unknown, monitoring ALDH2 dynamics during the development of acquired drug resistance may offer valuable mechanistic insights.

The interplay between ALDH2 (aldehyde metabolism) and MAPK/ERK (survival signal pathway) suggests a feedback control network, influencing tumor cell proliferation and treatment response. While low ALDH2 activity increases cellular susceptibility to AcAH toxicity, it may paradoxically confer malignant potential and survival advantage to tumor cells under drug pressure, suggesting that restoring ALDH2 activity could be a potential therapeutic strategy in melanoma patients undergoing targeted therapy. Supporting this hypothesis, we found that pharmacological activation of ALDH2 by Alda-1 attenuated AcAH-induced activation of MAPK/ERK and other MAPK signaling pathways. Additionally, *ALDH2*-OE in *ALDH2*-low 1205Lu cells not only reduced AcAH accumulation, oxidative stress, and inflammatory status but also enhanced mitochondrial function and promoted oxidative phosphorylation.

Furthermore, additional mechanisms beyond enhanced MAPK/ERK signaling may contribute to the role of ALDH2 in tumor growth and response to targeted therapy. Although ALDH isoforms exhibit substrate specificity, some overlap exists. For example, ALDH1 family members (including ALDH1A1, ALDH1A3, and ALDH1B1) and ALDH3A1 can also metabolize AcAH [61]. We analyzed the gene expression of ALDH1 isoforms in *ALDH2*-KO A375 and HT144 cells compared to their WT counterparts. *ALDH1A* expression was extremely low in both cell lines, rendering its interpretation limited. In contrast, following *ALDH2* loss, *ALDH1A3* was upregulated in HT144 cells, and *ALDH1B1* expression was increased in both A375 and HT144 cells (Figure S15). While these upregulated ALDH1 isoforms may serve as compensatory enzymes for AcAH detoxification, their physiological relevance is likely limited due to their lower affinity for AcAH [61]. The Km (Michaelis constant) values of ALDH1A1, ALDH1A3, and ALDH1B1 for AcAH are approximately 180, 2400, and 55 µM, respectively, corresponding to 900-, 12,000-, and 275-fold higher values compared to ALDH2, which has a Km of 0.2 µM [62,63]. ALDH1 isoforms are also known regulators of melanoma stemness [64,65]. It is plausible that ALDH2 downregulation is accompanied by compensatory ALDH1 upregulation, enhancing stemness and subsequent drug resistance, although this hypothesis remains untested. In addition, the p38/ATF2/PI3K/AKT pathway has been implicated in melanoma progression and therapeutic resistance [66]. We observed that AcAH activated p38 and ATF2, while treatment with Alda-1 suppressed such activation (Figure S16). These findings suggest that ALDH2 deficiency may contribute to tumor progression and resistance to targeted therapy through AcAH-mediated p38/ATF2 activation.

Crucially, more comprehensive in vivo studies, including xenograft models with *ALDH2*-KO and rescue experiments with *ALDH2*-OE or enzyme activators, are warranted to further validate the causal role of ALDH2 in tumor progression and therapeutic response. It is also important to note that the effects of *ALDH2* downregulation on tumor growth and drug response may be cell line-dependent and may not extend to *BRAF*-WT melanoma. Moreover, the implication of ALDH2 in tumorigenesis and therapeutic response is highly context-dependent. While ALDH2 suppresses tumor growth in melanoma [13], lung adenocarcinoma [48], and hepatocellular carcinoma [49], it has been shown to promote tumorigenesis in colorectal cancer [67].

In summary, our study highlights the critical role of ALDH2 in melanoma progression and therapy resistance. We demonstrate that ALDH2 downregulation enhances MAPK/ERK activation, promotes tumor growth, and confers resistance to BRAF and MEK inhibitors. Furthermore, we reveal that melanoma cells with acquired drug resistance exhibit further ALDH2 suppression, suggesting a potential feedback loop between ALDH2 and MAPK/ERK signaling. These findings suggest that targeting ALDH2 may provide a novel therapeutic approach for melanoma patients, particularly those undergoing MAPK/ERK-targeted therapy.

## 5. Conclusions

We demonstrate that *ALDH2* downregulation in melanoma is associated with increased tumor aggressiveness and resistance to targeted therapy. Understanding the mechanisms driving *ALDH2* downregulation provides new insights into melanoma progression and therapeutic resistance. Given that lower *ALDH2* expression correlates with poorer patient outcomes, our findings highlight *ALDH2* as a potential prognostic marker and therapeutic target in melanoma management.

## Data Availability

The data presented in this study are available in this article or associated Supplementary Material.

## Supplementary Materials

The following supporting information can be downloaded at: https://www.mdpi.com/article/10.3390/cells14120913/s1, Table S1: Primers used for qRT-PCR; Figure S1: Original blots of Figure 2c; Figure S2: Original blots of Figure 3a; Figure S3: Western blot analysis of the phosphorylation of ERK, p38, and JNK in A375 cells exposed to 5 mM AcAH for different hours; Figure S4: Western blot analysis of the phosphorylation of ERK, p38, and JNK in A375 cells pretreated with daidzin or Alda-1 for 2 h, followed by exposure to 5 mM AcAH for 1 h; Figure S5: Original blots of Figure 4b; Figure S6: Original blots of Figure 4c; Figure S7: *ALDH2* downregulation in *ALDH2*-normal HT144 led to increased ERK activation; Figure S8: Effects of *ALDH2* downregulation on *ATF4*, *IL1B*, *MITF* and *MYC* expression in *ALDH2*-normal HT144 cells; Figure S9: Original blots of Figure 5a; Figure S10: Original blots of Figure 6c; Figure S11: Original blots of Figure 6e; Figure S12: MTS proliferation assay of WT and *ALDH2*-OE 1205Lu cells treated with one dose of VEM and/or TRA for 48 h; Figure S13: Original blots of Figure 7; Figure S14: Inhibition of BRAF and MEK leads to increased ALDH2 expression in parental melanoma cells; Figure S15: Effects of *ALDH2* downregulation on *ALDH1A1*, *ALDH1A3*, and *ALDH1B1* expression in A375 and HT144 cells; and Figure S16: Western blot analysis of the phosphorylation of p38, ATF2 and AKT in HS294T cells pretreated with 20 μM daidzin or Alda-1 for 2 h, followed by exposure to 5 mM AcAH for 1 h.
