# Supplementary material for: Downregulated ALDH2 Contributes to Tumor Progression and Targeted Therapy Resistance in Human Metastatic Melanoma Cells
Source: Cells. 2025 Jun 17;14(12):913. doi: 10.3390/cells14120913 (PMC12191128; doi:10.3390/cells14120913)
Supplement: Supplementary file 1 [file cells-14-00913-s001.zip › cells-3618815 supplementary.pdf]

Supplemental data

Table S1. Primers used for qRT-PCR.

Gene	Gene ID	Forward	Reverse
ALDH2	217	GGGACAAGGAAGATGTGGACAA	AGGAGATGACATAGGGCTTGC
ATF4	468	ACCCAATTGGCCATCTCCCAGAA	ATCCACTTCACTGCCCAGCTCTAAAC
IL1B	3553	CAGGCTGCTCTGGGATTCTC	GTCCTGGAAGGAGCACTTCAT
MITF	4286	CAGGCATGAACACACATTCAC	TCCATCAAGCCCAAGATTTC
MITF-M	4286	GGAGCTCACAGCGTGTATTTTTCC	GACTTGGGAATCAAAGTACCTAGTTC
MYC	4609	TTCGGGTAGTGGAAAACCAG	AGTAGAAATACGGCTGCACC
ALDH1A1	216	ACTGCTCTCCACGTGGCATCTTTA	TGCCAACCTCTGTTGATCCTGTGA
ALDH1A3	220	ACCTGGAGGTCAAGTTCACCAAGA	ACGTCGGGCTTATCTCCTTCTTCC
ALDH1B1	219	CAGGTGGACAAGGAGCAGTTTG	ACGCCACCAAAGACAGTAGGCT
GAPDH	2597	CAGGGCTGCTTTTAACTCTGG	TGGGTGGAATCATATTGGAACA

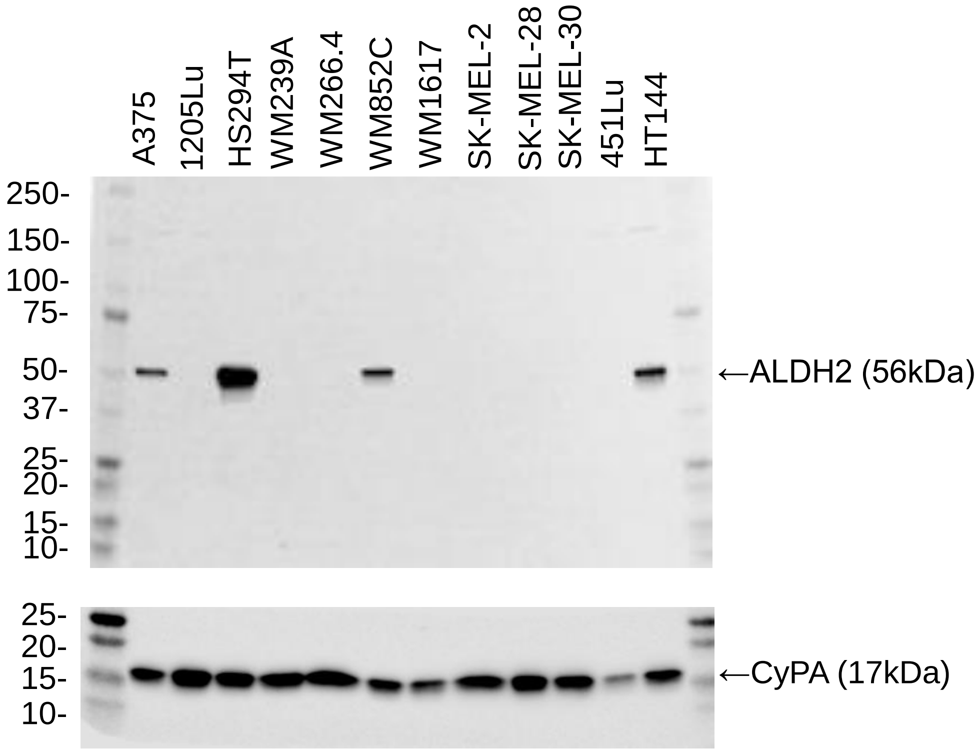

Figure S1. Original blots of Figure 2c.

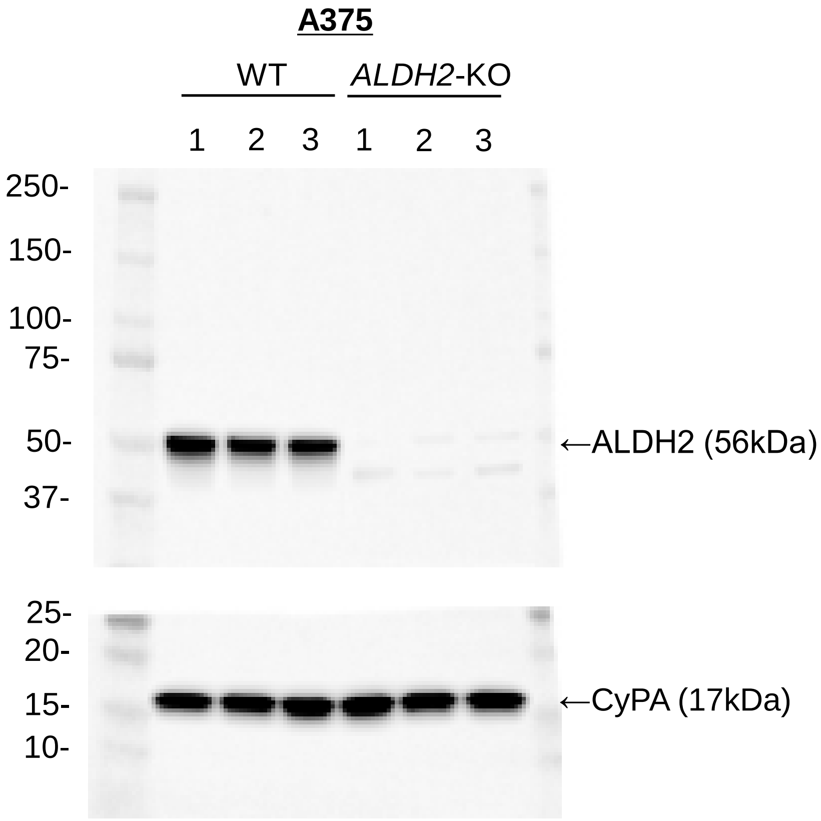

Figure S2. Original blots of Figure 3a.

A375 (*ALDH2*-WT cells)

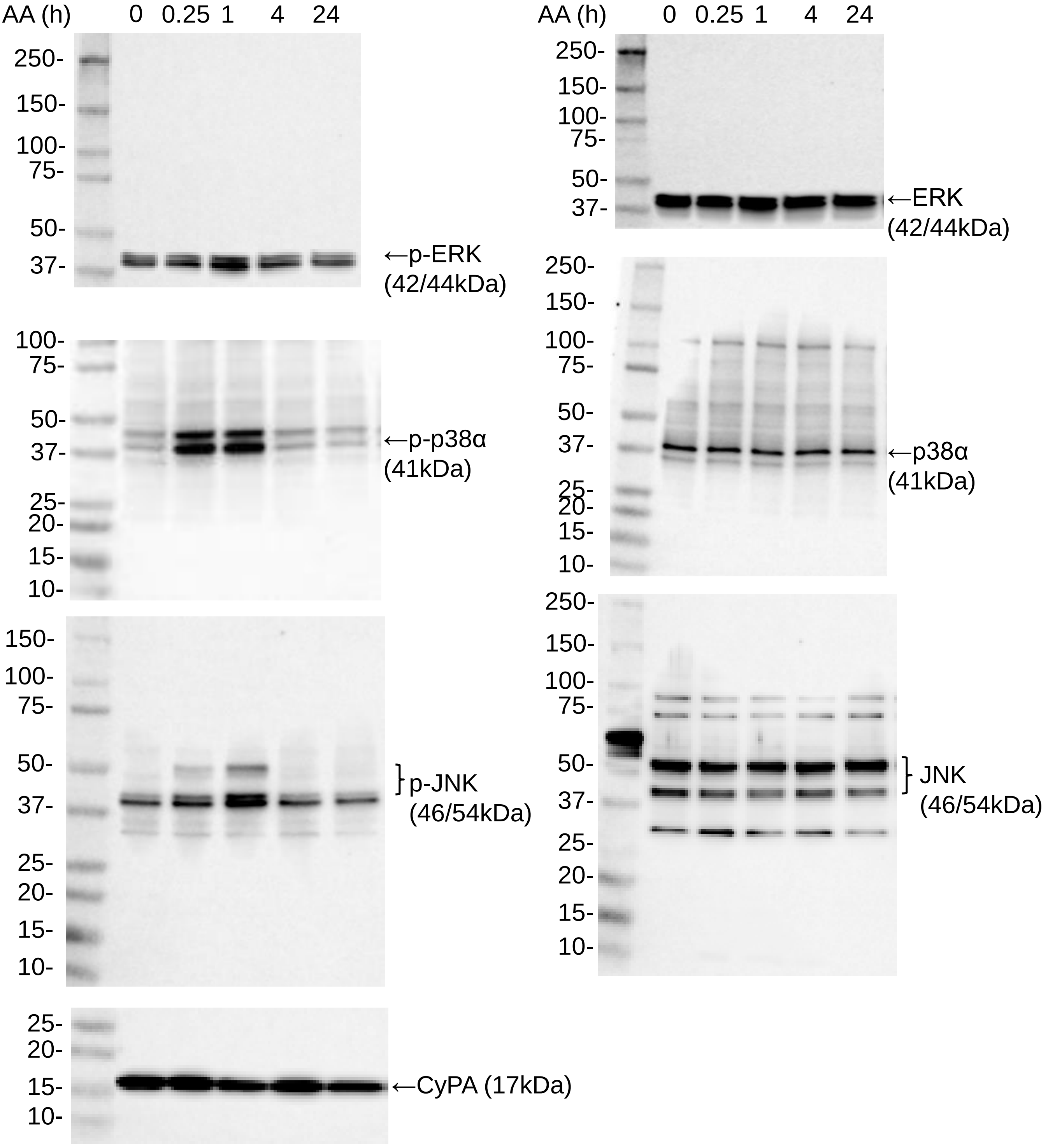

Figure S3. Western blot analysis of the phosphorylation of ERK, p38, and JNK in WT A375 cells exposed to 5 mM AcAH for different hours.

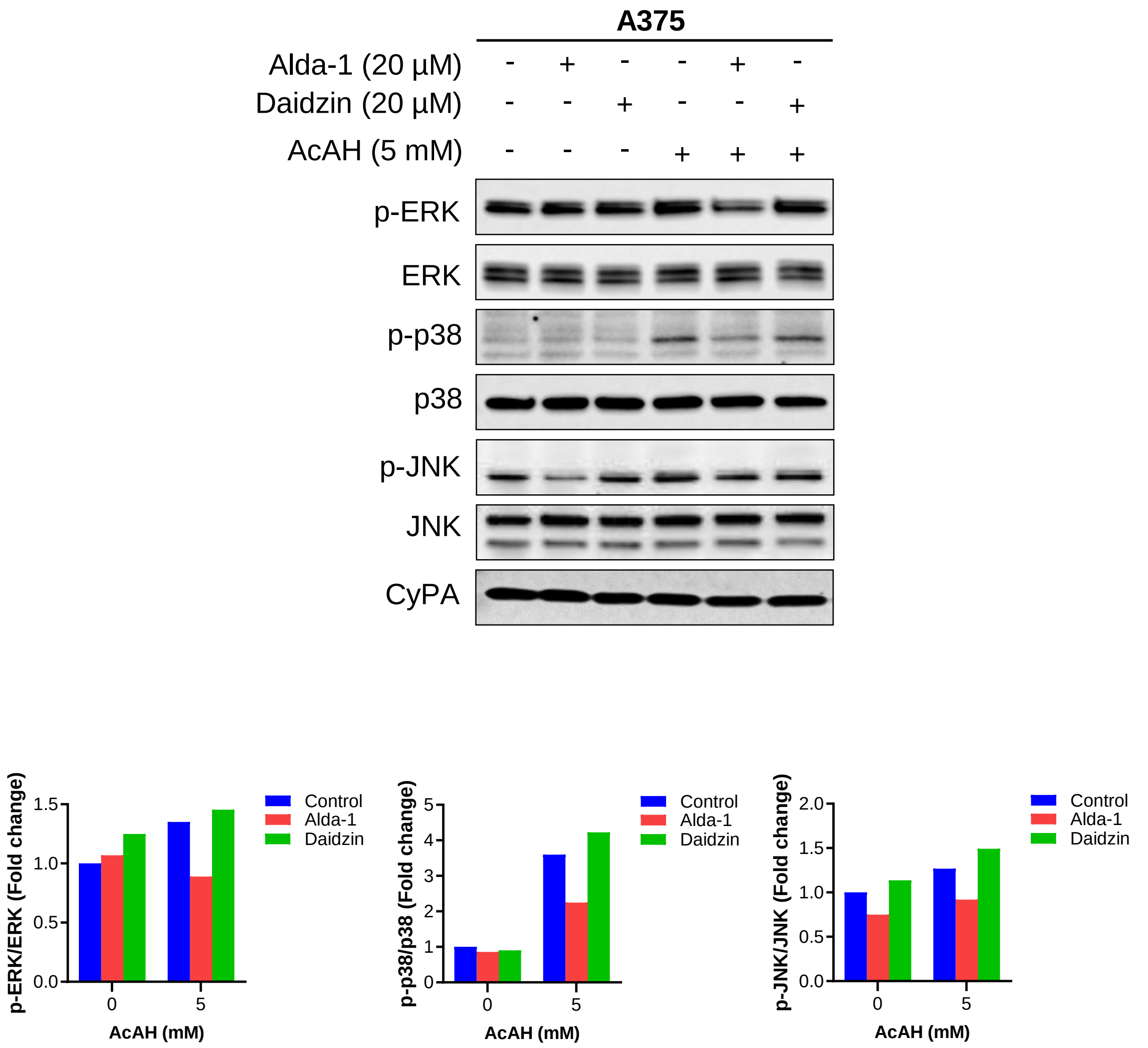

Figure S4. Western blot analysis of the phosphorylation of ERK, p38, and JNK in A375 cells pretreated with daidzin or Alda-1 for 2 h, followed by exposure to 5 mM AcAH for 1 h. Note that JNK quantification was based on the 46 kDa band.

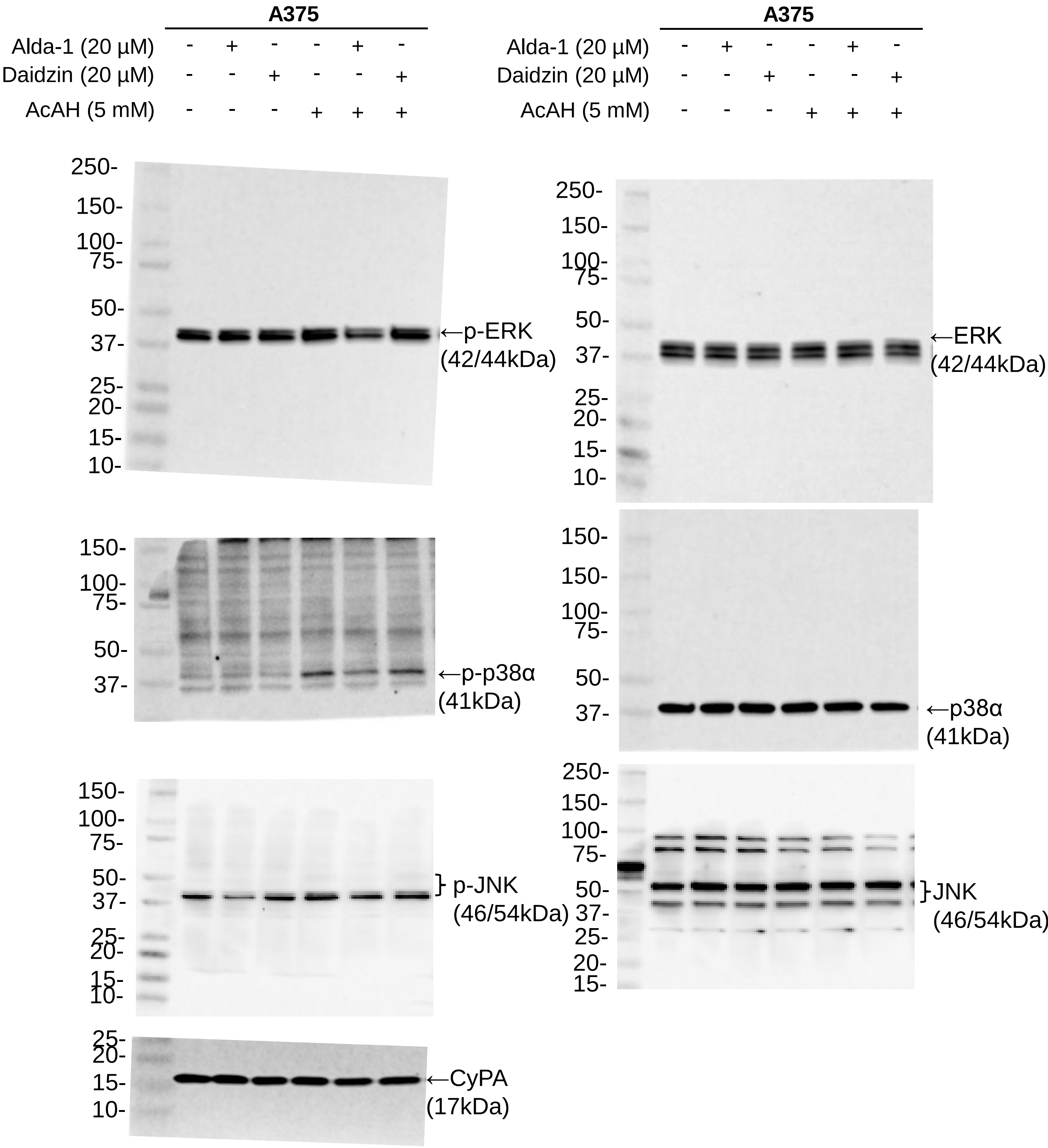

Figure S4. (Cont'd).

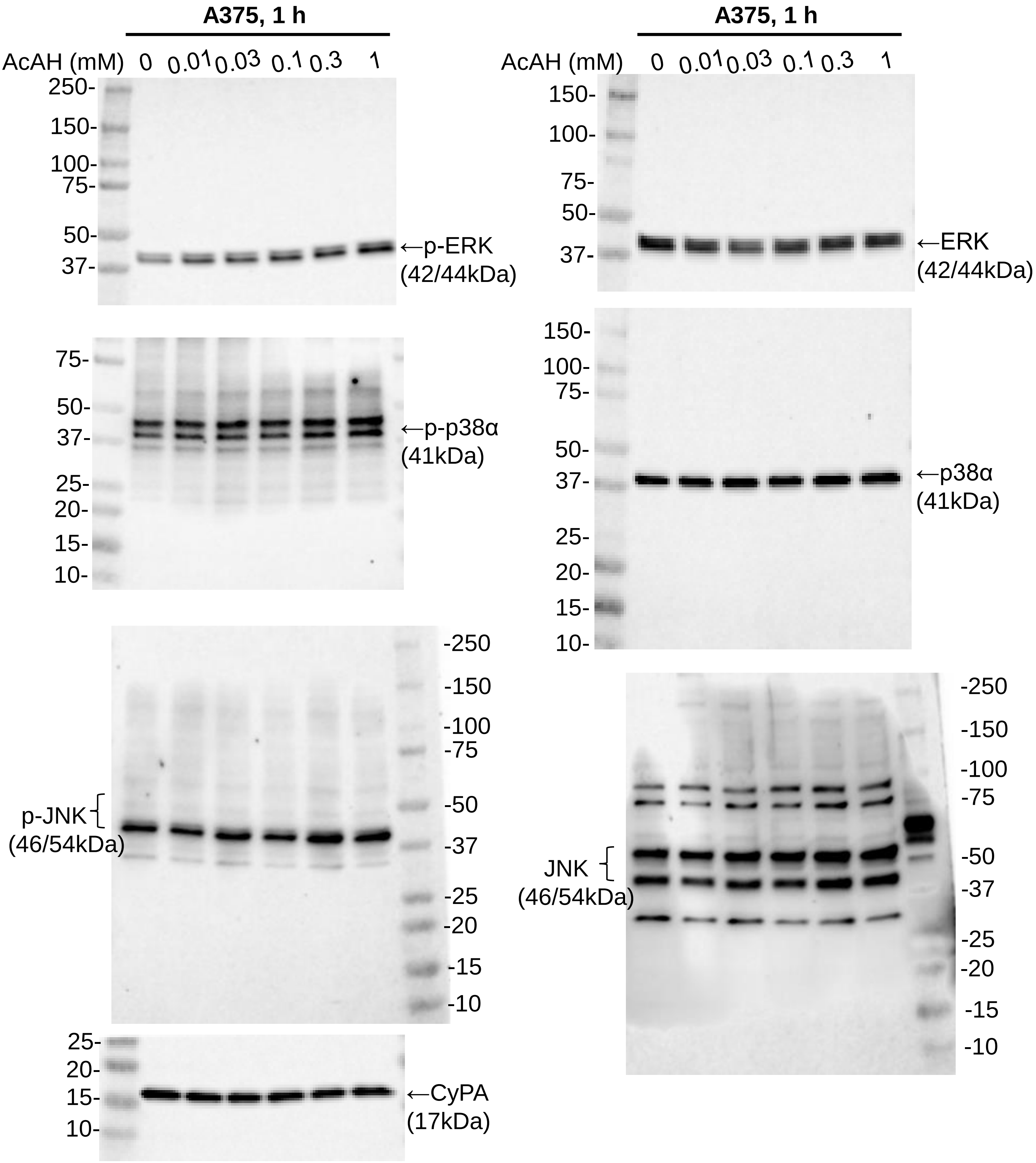

Figure S5. Original blots of Figure 4b.

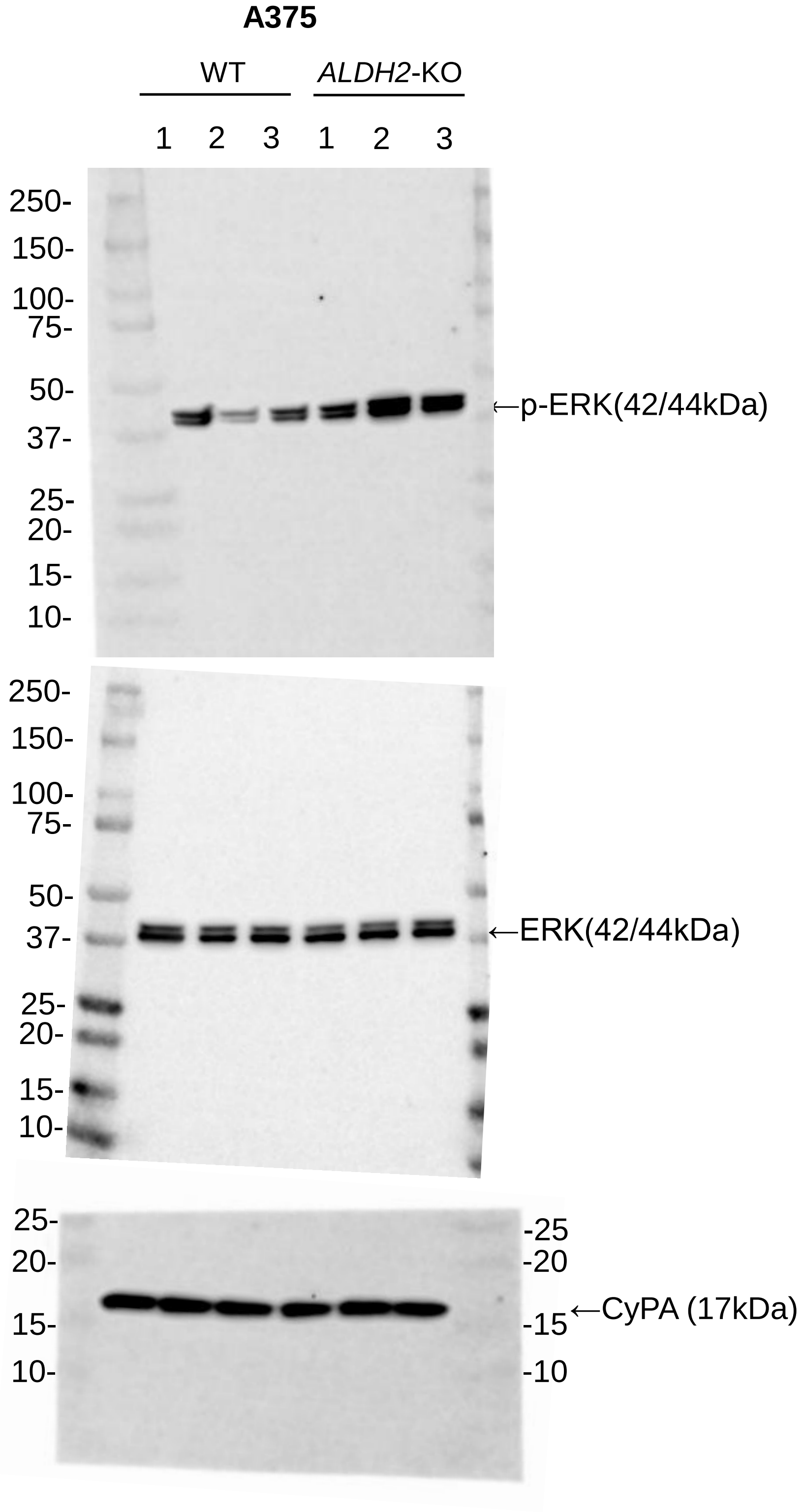

Figure S6. Original blots of Figure 4c.

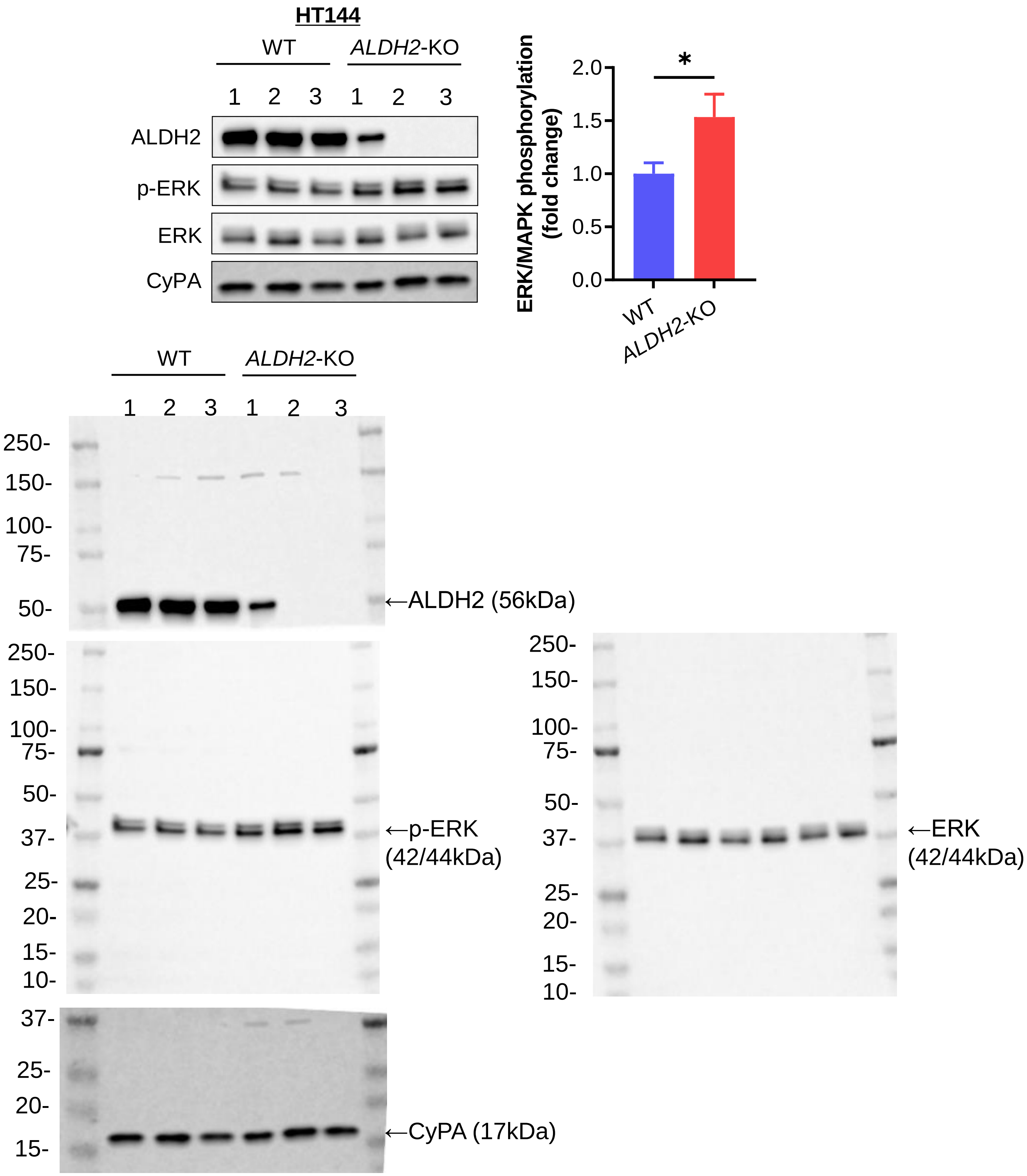

Figure S7. *ALDH2* downregulation in *ALDH2*-normal HT144 led to increased ERK activation. Western blot analysis of ALDH2 and baseline phosphorylation levels of ERK/MAPK in WT and *ALDH2*-KO HT144 cells. Band densities of phosphorylated ERK were quantified and adjusted. The data are expressed as the mean ± SD (n = 3 (WT) or 2 (KO #2 and 3)). *P < 0.05.

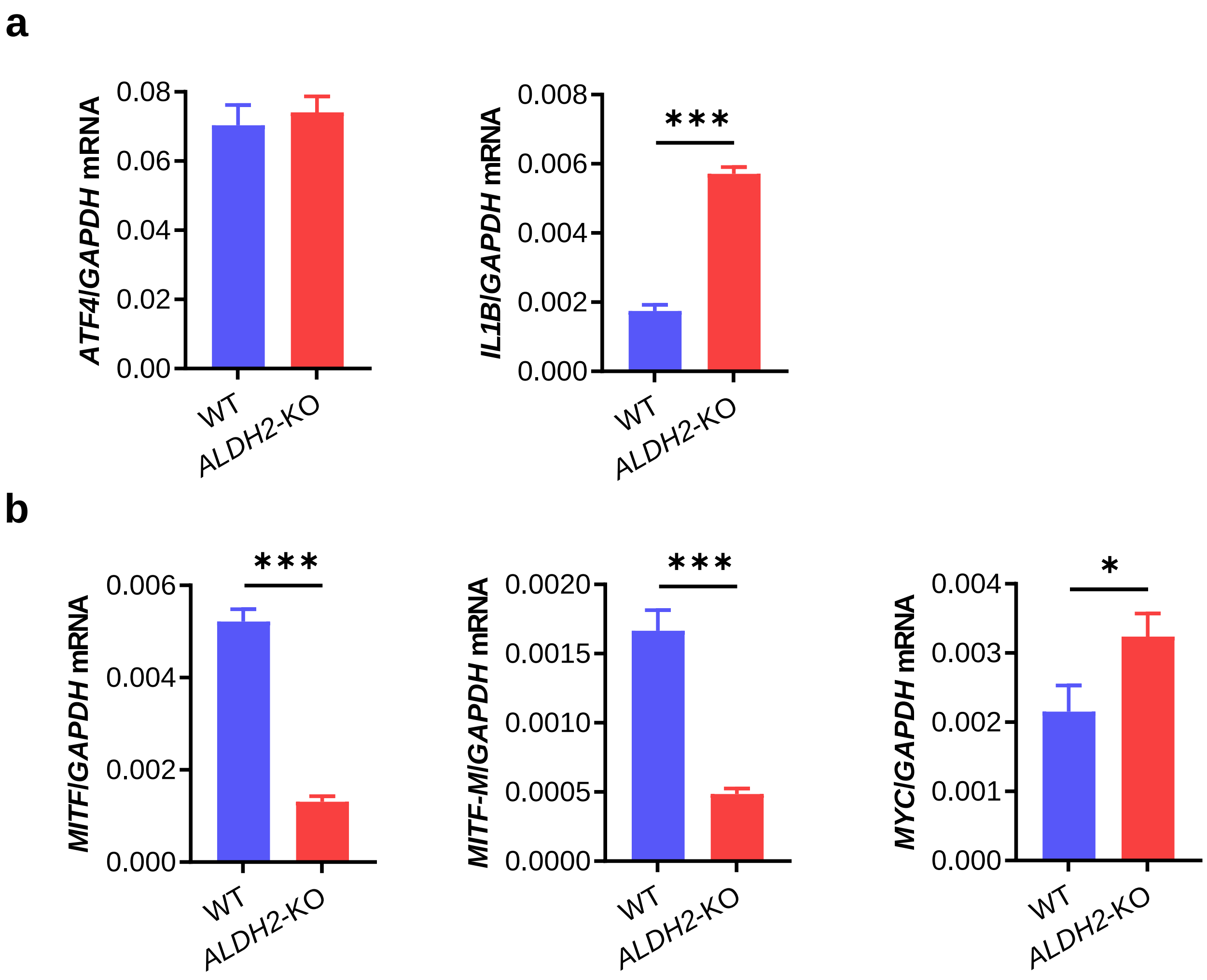

Figure S8. Effects of *ALDH2* downregulation on *ATF4*, *IL1B*, *MITF* and *MYC* expression in *ALDH2*-normal HT144 cells. (**a**) qRT-PCR of *ATF4* and *IL1B* mRNA. (**b**) qRT-PCR of *MITF*, *MITF-M*, and *MYC* mRNA. The data are expressed as the mean ± SD (n = 3). *P < 0.05 and ***P < 0.001.

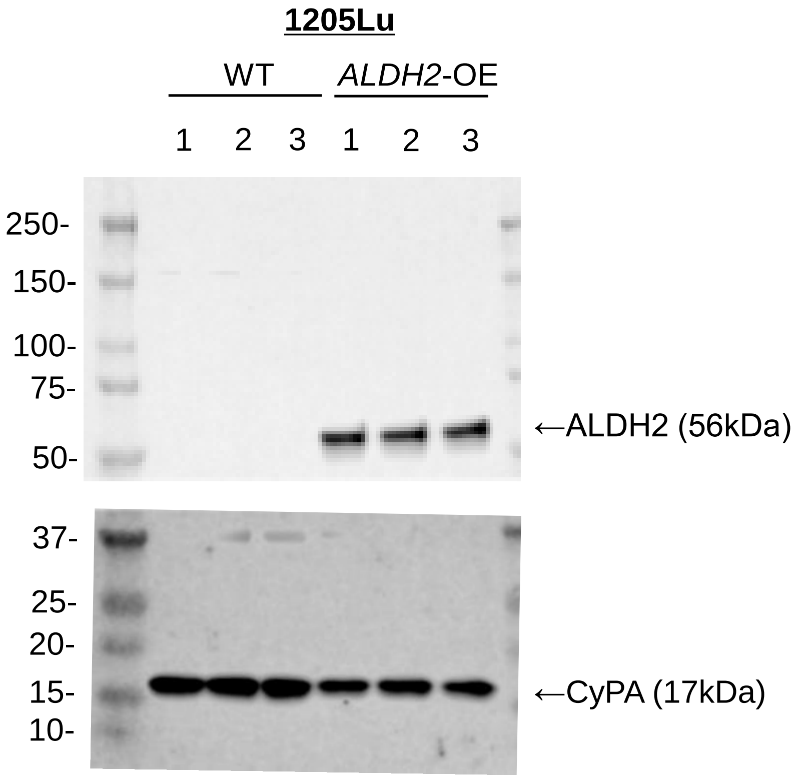

Figure S9. Original blots of Figure 5a.

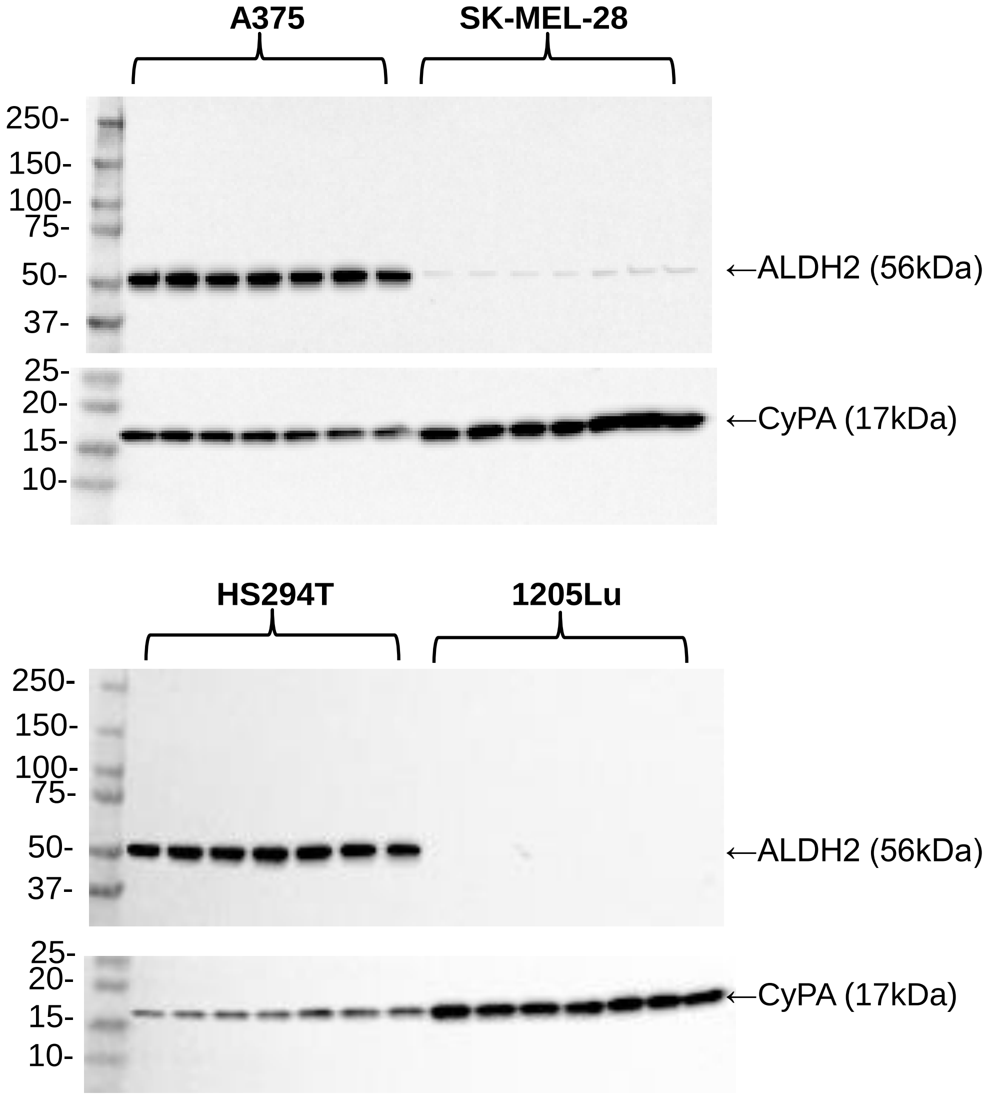

Figure S10. Original blots of Figure 6c. HS294T: BRAF wild-type melanoma cell line.

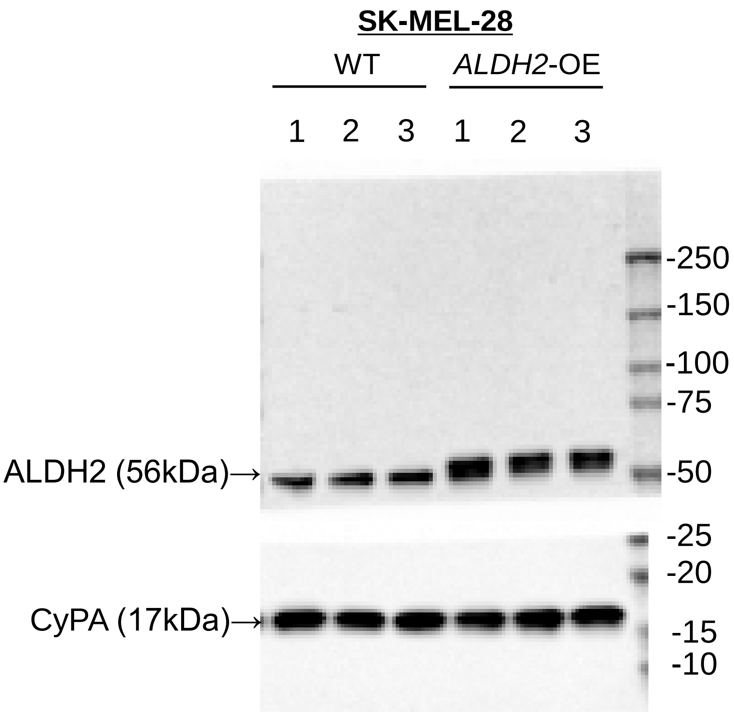

Figure S11. Original blots of Figure 6e.

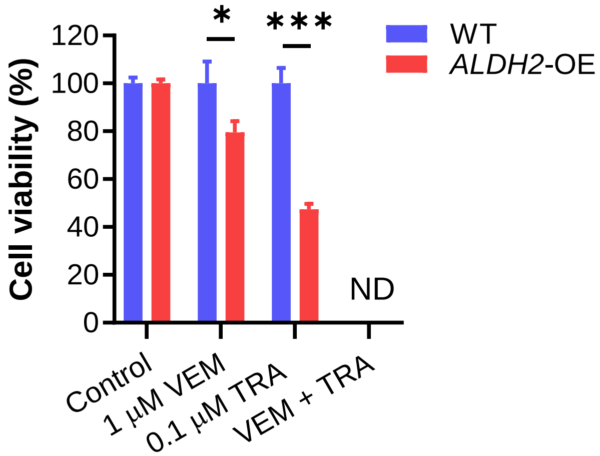

Figure S12. MTS proliferation assay of WT and *ALDH2*-OE 1205Lu cells treated with one dose of VEM and/or TRA for 48 h. For comparison, WT cells were adjusted to 100%. ND, not determined. The data are expressed as the mean ± SD (n = 4). *P < 0. 05 and ***P < 0. 001.

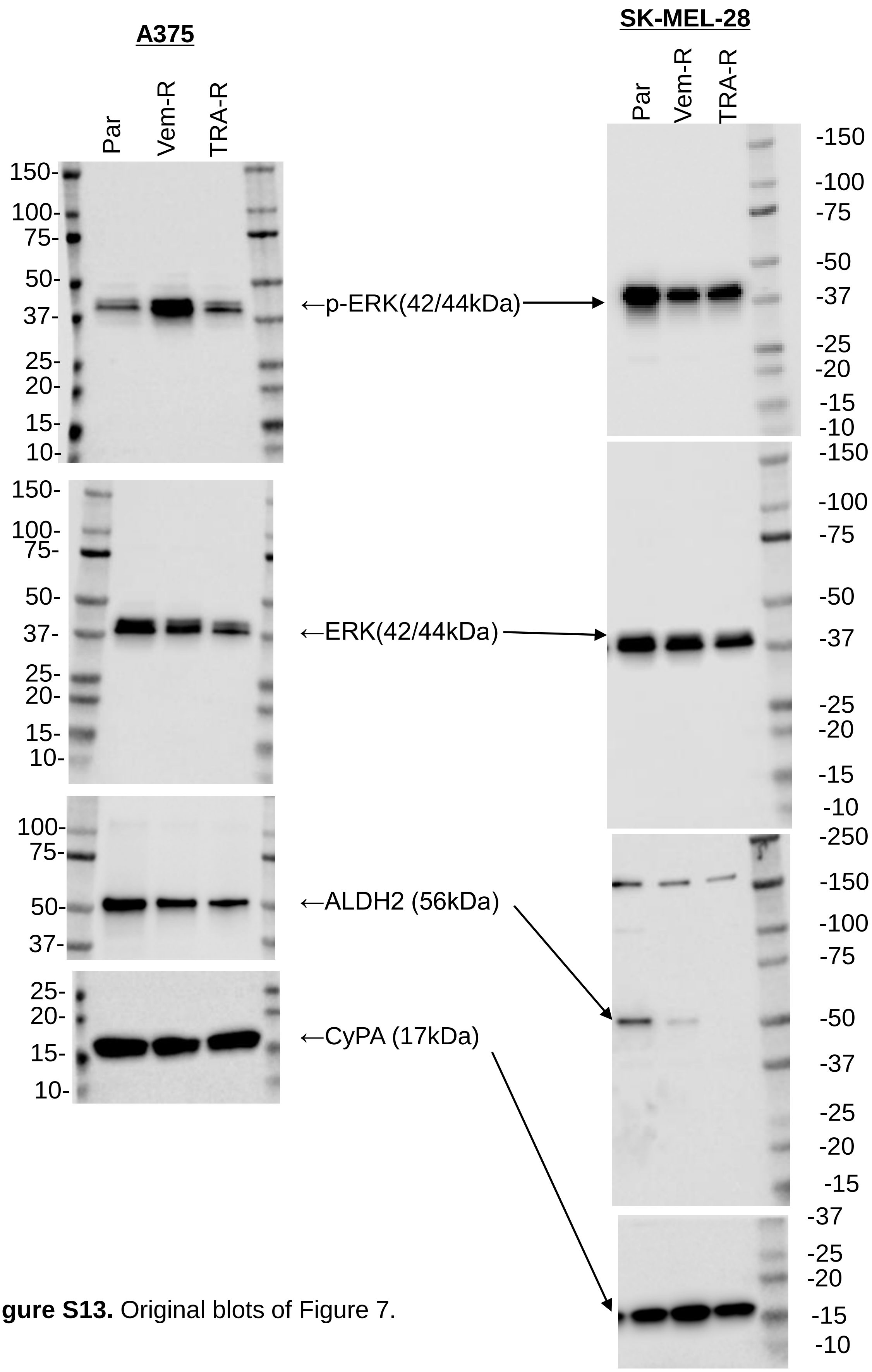

Figure S13. Original blots of Figure 7.

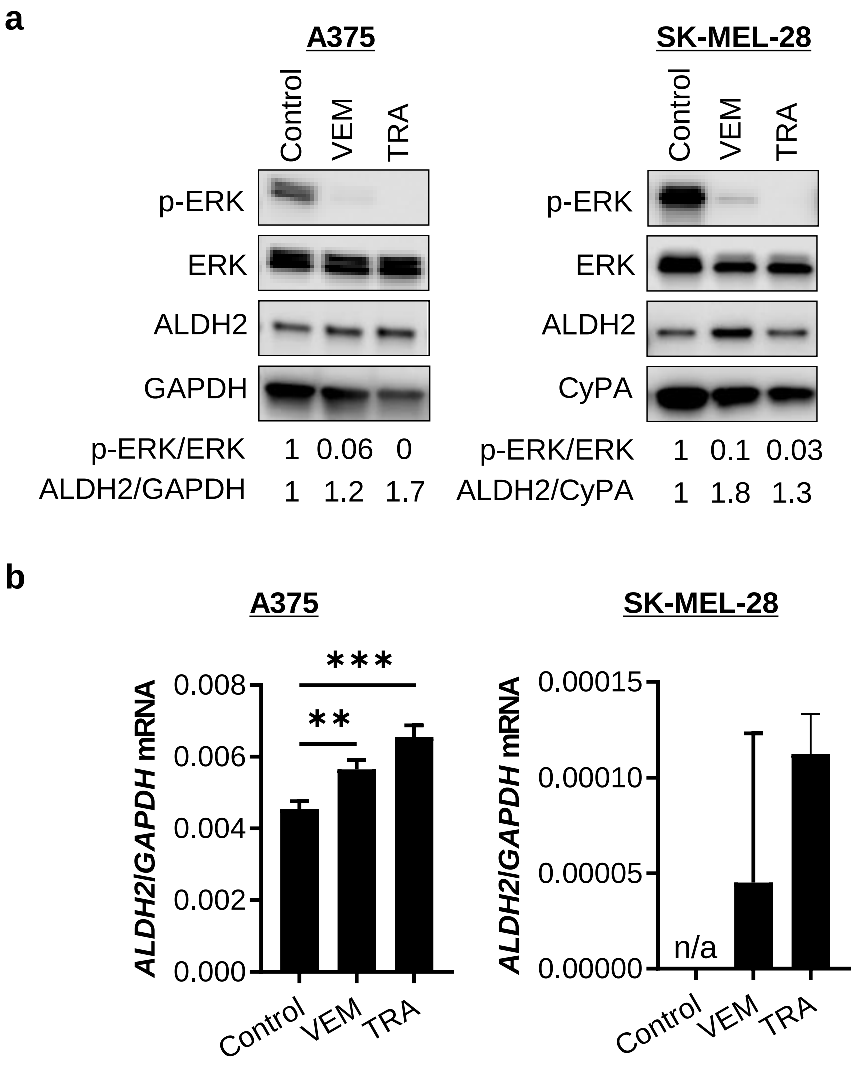

Figure S14. Inhibition of BRAF and MEK leads to increased ALDH2 expression in parental melanoma cells. (**a**) Western blot analysis of ERK phosphorylation and ALDH2 expression in A375 and SK-MEL-28 cells treated with dimethyl sulfoxide (control), 1 μM VEM or 0.1 μM TRA for 20 h. The band densities of p-ERK and ALDH2 were quantitated and normalized. (**b**) Quantitative RT-PCR of *ALDH2* mRNA in A375 and SK-MEL-28 cells treated with VEM or TRA for 20 h. Mean ± SD (n = 3). **P < 0. 01 and ***P < 0. 001.

A375 (parental cells)

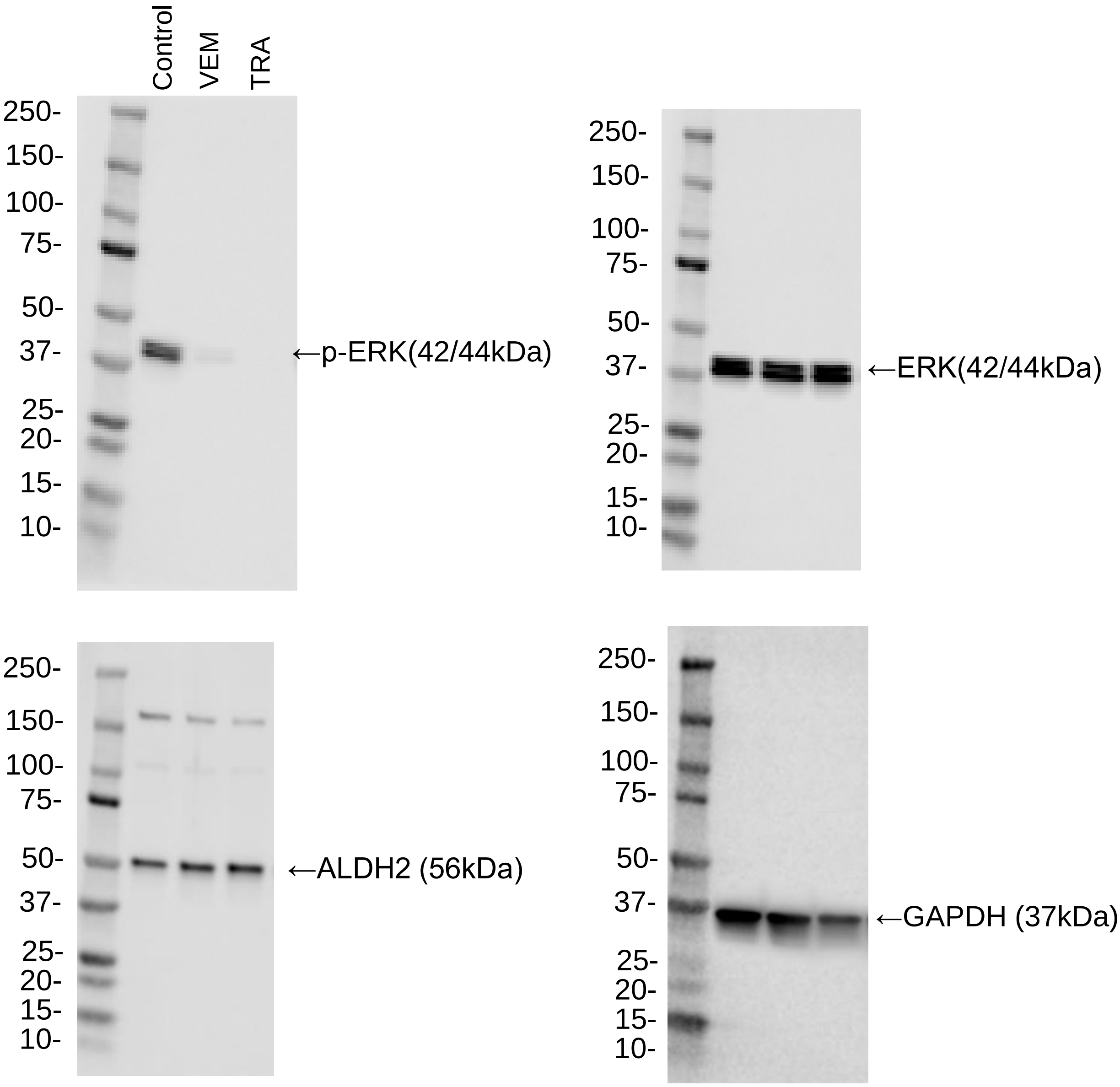

Figure S14. (Cont'd).

SK-MEL-28

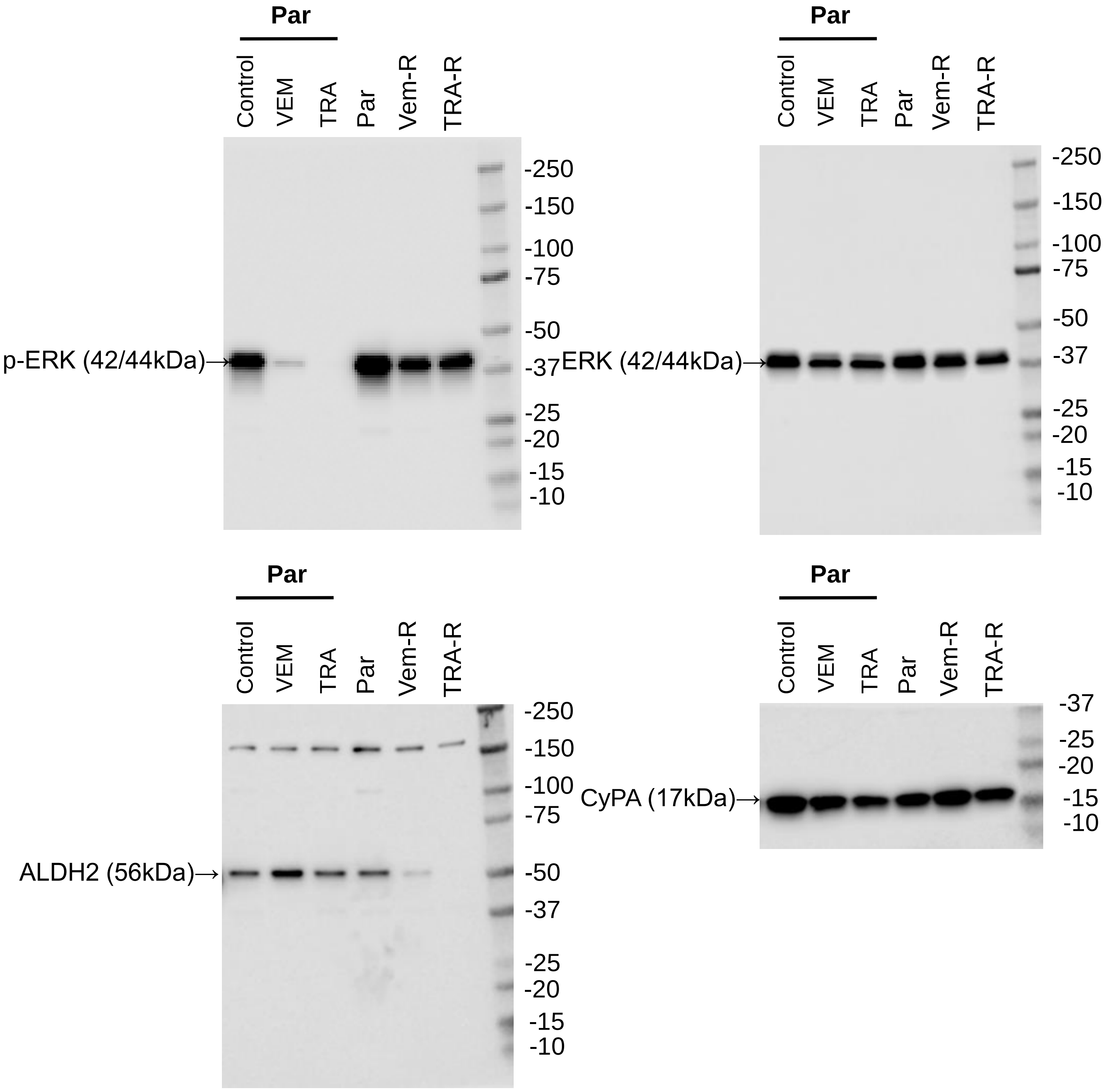

Figure S14. (Cont’d. Note that resistant cells are used in **Figure S13**).

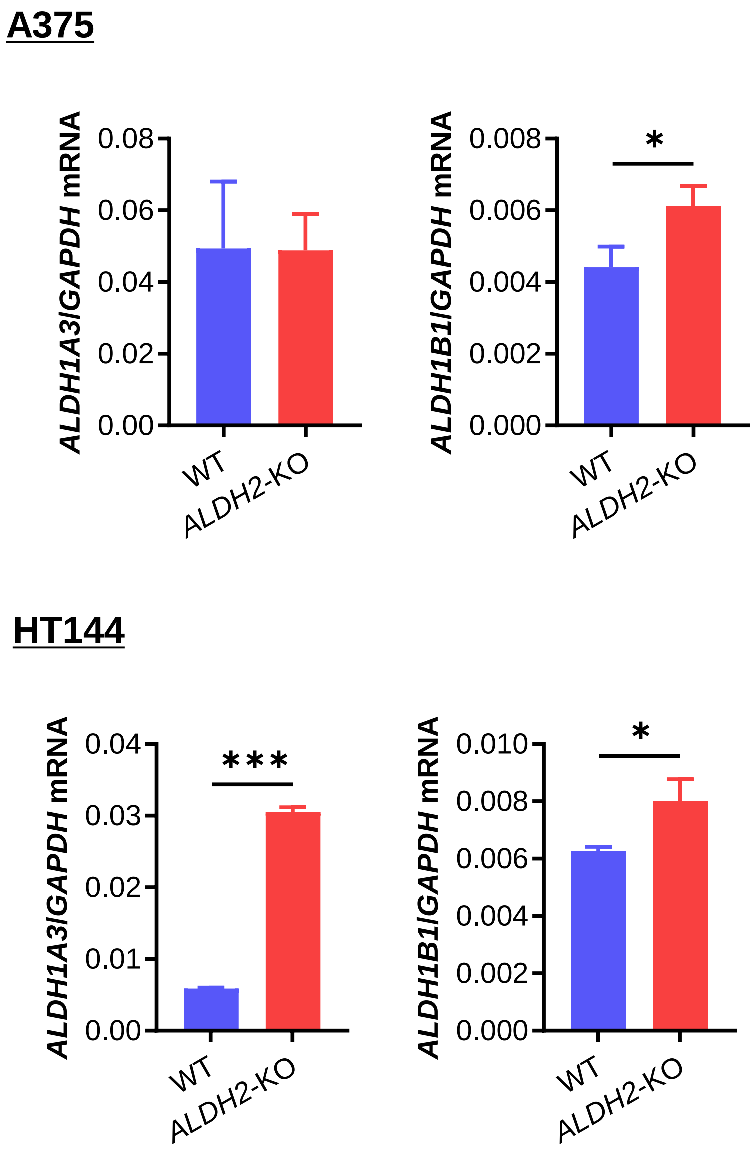

Figure S15. Effects of *ALDH2* downregulation on *ALDH1A1*, *ALDH1A3,* and *ALDH1B1* expression in A375 and HT144 cells. *ALDH1A1* expression levels were extremely low in both cell lines and are not shown here. The data are expressed as the mean ± SD (n = 6 for A375 and 3 for HT144). *P < 0.05 and ***P < 0.001.

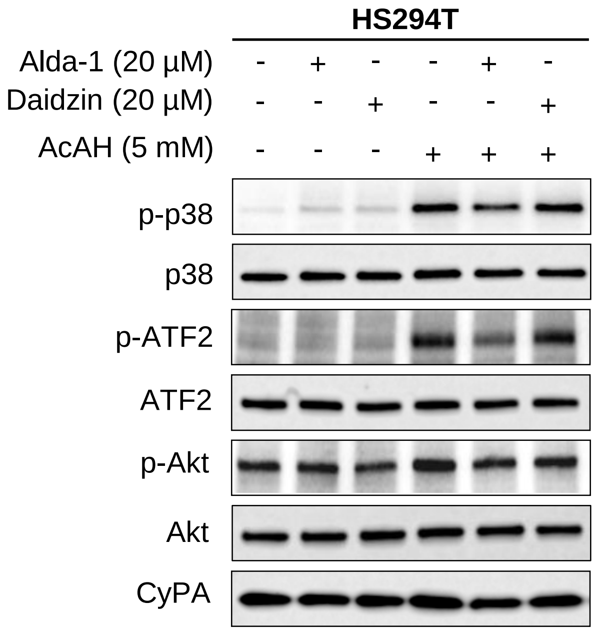

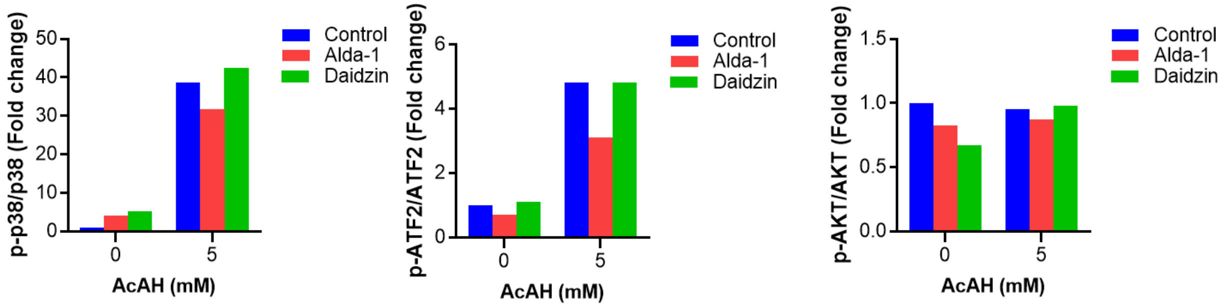

Figure S16. Western blot analysis of the phosphorylation of p38, ATF2 and AKT in HS294T cells pretreated with 20 µM daidzin or Alda-1 for 2 h, followed by exposure to 5 mM AcAH for 1 h.

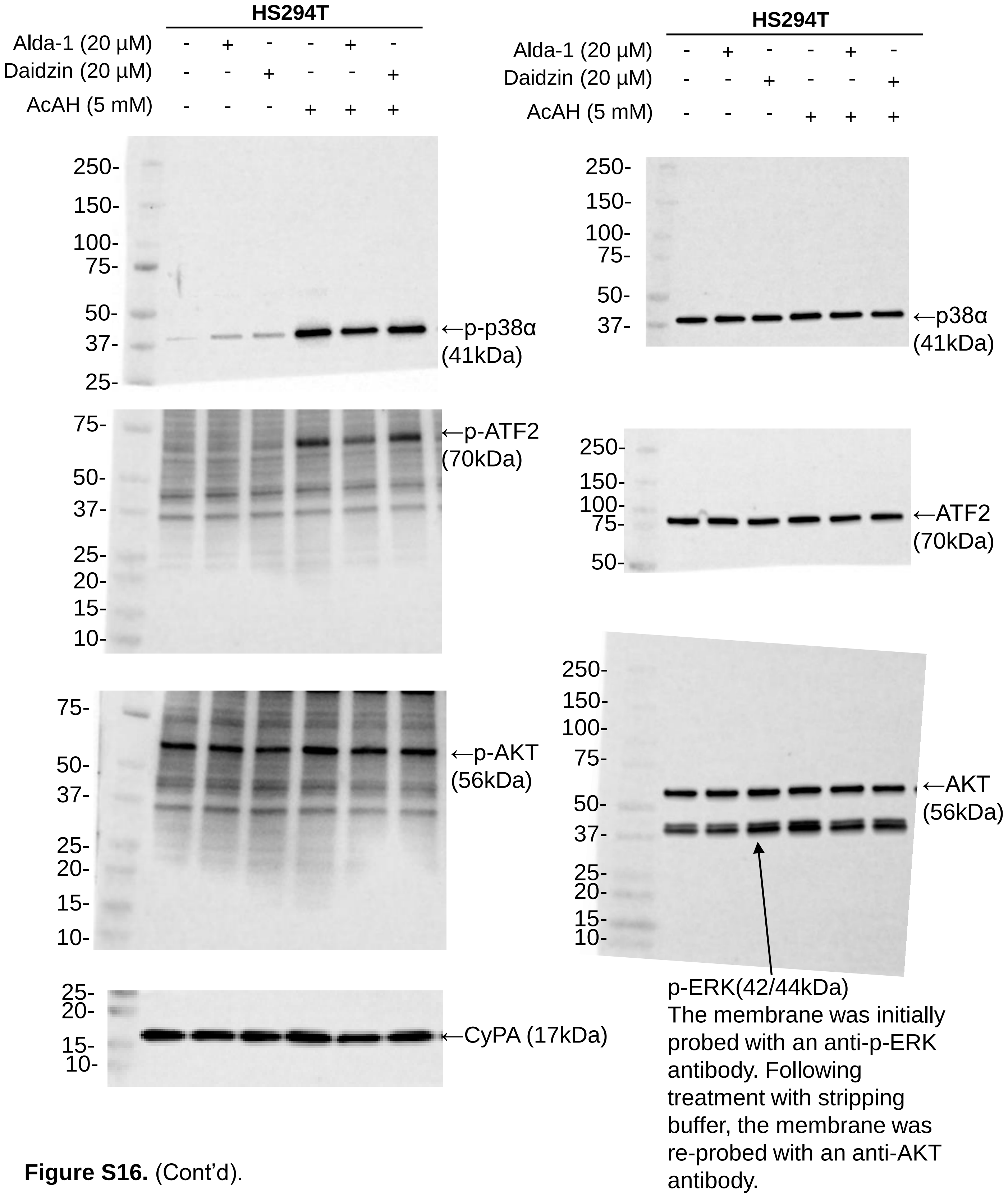

Figure S16. (Cont'd).
